# Apomictic and Sexual Germline Development Differ with Respect to Cell Cycle, Transcriptional, Hormonal and Epigenetic Regulation

**DOI:** 10.1371/journal.pgen.1004476

**Published:** 2014-07-10

**Authors:** Anja Schmidt, Marc W. Schmid, Ulrich C. Klostermeier, Weihong Qi, Daniela Guthörl, Christian Sailer, Manuel Waller, Philip Rosenstiel, Ueli Grossniklaus

**Affiliations:** 1 Institute of Plant Biology & Zürich-Basel Plant Science Center, University of Zürich, Zürich, Switzerland; 2 Institute of Clinical Molecular Biology, Christian-Albrechts University, Kiel, Germany; 3 Functional Genomics Center Zürich, UZH/ETH Zürich, Zürich, Switzerland; CCMB, United States of America

## Abstract

Seeds of flowering plants can be formed sexually or asexually through apomixis. Apomixis occurs in about 400 species and is of great interest for agriculture as it produces clonal offspring. It differs from sexual reproduction in three major aspects: (1) While the sexual megaspore mother cell (MMC) undergoes meiosis, the apomictic initial cell (AIC) omits or aborts meiosis (apomeiosis); (2) the unreduced egg cell of apomicts forms an embryo without fertilization (parthenogenesis); and (3) the formation of functional endosperm requires specific developmental adaptations. Currently, our knowledge about the gene regulatory programs underlying apomixis is scarce. We used the apomict *Boechera gunnisoniana*, a close relative of *Arabidopsis thaliana*, to investigate the transcriptional basis underlying apomeiosis and parthenogenesis. Here, we present the first comprehensive reference transcriptome for reproductive development in an apomict. To compare sexual and apomictic development at the cellular level, we used laser-assisted microdissection combined with microarray and RNA-Seq analyses. Conservation of enriched gene ontologies between the AIC and the MMC likely reflects functions of importance to germline initiation, illustrating the close developmental relationship of sexuality and apomixis. However, several regulatory pathways differ between sexual and apomictic germlines, including cell cycle control, hormonal pathways, epigenetic and transcriptional regulation. Enrichment of specific signal transduction pathways are a feature of the apomictic germline, as is spermidine metabolism, which is associated with somatic embryogenesis in various plants. Our study provides a comprehensive reference dataset for apomictic development and yields important new insights into the transcriptional basis underlying apomixis in relation to sexual reproduction.

## Author Summary

In flowering plants, asexual reproduction through seeds (apomixis) likely evolved from sexual ancestors several times independently. Only three key developmental steps differ between sexual reproduction and apomixis. In contrast to sexual reproduction, in apomicts the first cell of the female reproductive lineage omits or aborts meiosis (apomeiosis) to initiate gamete formation. Subsequently, the egg cell develops into an embryo without fertilization (parthenogenesis), and endosperm formation can either be autonomous or depend on fertilization. Consequently, the offspring of apomicts is genetically identical to the mother plant. The production of clonal seeds bears great promise for agricultural applications. However, the targeted manipulation of reproductive pathways for seed production has proven difficult as knowledge about the underlying gene regulatory processes is limited. We performed cell type-specific transcriptome analyses to study apomictic germline development in *Boechera gunnisoniana*, an apomictic species closely related to *Arabidopsis thaliana*. To facilitate these analyses, we first characterized a floral reference transcriptome. In comparison, we identified several regulatory pathways, including core cell cycle regulation, protein degradation, transcription factor activity, and hormonal pathways to be differentially regulated between sexual and apomictic plants. Apart from new insights into the underlying transcriptional networks, our dataset provides a valuable starting point for functional investigations.

## Introduction

In flowering plants, both sexual and asexual reproduction through seeds (apomixis) is common. Apomixis occurs in more than 400 plant species belonging to over 40 families, but it is poorly represented in crop species. Apomixis leads to clonal offspring by conservation of the maternal genotype through the absence of meiosis and fertilization [1]–[4]. Engineering of apomixis in crop species is perceived as one of the greatest challenges faced by modern agriculture [5]. However, achieving this goal proved to be difficult, particularly as the knowledge about the genetic basis and regulatory programs underlying apomictic reproduction is very limited.

Sexual reproduction and apomixis only differ in a number of key developmental steps [6], [7]. During sexual reproduction, the female and male reproductive lineages are initiated by spore formation from a spore mother cell during megasporogenesis and microsporogenesis, respectively. The megaspore mother cell (MMC) is the first cell of the female germline. It is specified by selection of one subepidermal, somatic (sporophytic) cell within an ovule, the precursor of the seed. The MMC undergoes meiosis and gives rise to a tetrad of reduced megaspores. Typically, only one of those - the functional megaspore (FMS) - survives to form the female gametophyte (embryo sac). The FMS divides mitotically and subsequently cellularizes to form the mature female gametophyte harbouring the gametes (egg cell and central cell) and several accessory cells, including the synergids that play an important role in fertilization [8]. Double fertilization of the egg and central cell with one sperm each initiates the development of embryo and endosperm, respectively. In contrast, in gametophytic apomixis an unreduced sporophytic cell of the ovule in proximity to the MMC (apospory), or the MMC itself becoming an apomictic initial cell (AIC) that omits or aborts meiosis (diplospory), gives rise to an unreduced embryo sac (apomeiosis) [9]. The egg cell subsequently develops into an embryo without fertilization (parthenogenesis). Endosperm development can either be autonomous or require fertilization (pseudogamy). It is likely that signals from sporophytic ovule tissues are important for the development of the sexual and apomictic germline [6], [9]. During meiosis the MMC is shielded by incorporation of callose into its cell wall [10], which may temporarily reduce or block such signaling. However, to our knowledge such signaling events have so far not been investigated in detail.

While recent studies uncovered the transcriptional basis of key steps of female germline development in the sexual model species *Arabidopsis thaliana* [11]–[13], relatively little is known about the genetic and transcriptional basis governing apomictic reproduction. Gametophytic apomixis is genetically controlled by usually two or more loci - or potentially clusters of linked loci - in different aposporous and diplosporous species [14]–[24]. In the *Boechera* genus, there is evidence for a complex genetic control of apomixis [25]. At the transcriptional level it has been hypothesized that apomixis is derived from a deregulation of the sexual pathway [6], [7], [26]. Indeed, evidence for differential regulation of many genes between apomictic and sexual accessions comes from comparative gene expression analyses. These studies mostly use ovule or flower tissues from a variety of species, including *Boechera* spp. [27], [28], *Brachiaria* spp. [29], [30], *Hieracium perforatum* [31], *Pennisetum* spp. [32], [33], *Paspalum* spp. [34]–[36], apomeiotic mutants of *Medicago falcata* [37], *Panicum maximum* [38], and *Poa pratensis* [39], [40]. In addition, recent findings indicate spatial and temporal shifts in the expression of genes important for reproductive development between sexual and apomictic plants [27]–[29], [41].

To coordinate such complex transcriptional deregulation, the involvement of epigenetic regulatory pathways has been proposed [3], [6], [7]. Epigenetic pathways play important roles in regulating developmental and cell-fate decisions through the modification of gene activity by histone modifications, DNA methylation or gene silencing by small RNAs. Interestingly, features of apospory or diplospory have recently been observed in *Arabidopsis* and maize carrying mutant alleles of genes involved in DNA methylation and small RNA pathways [42]–[44]. In *Arabidopsis* plants carrying mutations in *ARGONAUTE9* (*AGO9*), or genes encoding additional members of a small RNA pathway (*RNA-DEPENDENT RNA POLYMERASE 6* (*RDR6*), *SUPPRESSOR OF GENE SILENCING 3* (*SGS3*)), additional MMC-like cells in the ovule gave rise to developing female gametophytes in a process resembling apospory [42]. Maize plants with mutations in homologues of the *Arabidopsis* DNA methyltransferases *DOMAINS REARRANGED METHYLASE1* (*DRM1*)/*DRM2* and *CHROMOMETHYLTRANSFERASE3* (*CMT3*) show also features of apospory [43]. However, in maize plants carrying mutations in *AGO104*, a homologue of *Arabidopsis AGO9*, formation of unreduced viable gametes occurs by a diplospory-like mechanism [44].

In addition, features of apospory have been observed in *Arabidopsis* plants carrying mutations in the RNA helicase gene *MNEME* (*MEM*), which restricts germline fate to one cell per ovule [12]. As in *ago9* mutants, the additional MMC-like cells initiate development of unreduced female gametophytes [12]. Apomeiosis has also been achieved by mutating important meiotic genes in *Arabidopsis*, such as *DYAD*/*SWITCH1* (*SWI1*), a regulator of meiotic chromosome organisation, or a combination of three mutations in the MiMe triple mutant (*sporulation11-1* (*spo11-1*); *omission of second division1* (*osd1*); *recombination8* (*rec8*)) [45], [46]. However, to date the potential role of these genes in naturally occurring apomixis has not been elucidated.

To study the transcriptional basis of key steps of apomictic reproduction we used the triploid, diplosporous species *Boechera gunnisoniana* as an apomictic model. The genus *Boechera* is closely related to the sexual model species *Arabidopsis thaliana*, facilitating comparative studies. We demonstrate the obligate apomictic behaviour of *B. gunnisoniana* by analysing the ploidy of embryo and endosperm in single seeds by means of a flow cytometric seed screen [47]. As no annotated, genome-wide sequence information is available for this species, we used RNA-Seq (Illumina HiSeq2000) to generate a reference transcriptome based on ovule tissues isolated by microdissection at the developmental stages of interest. We annotated the reference transcriptome, including the identification of homologous genes in *Arabidopsis*. Using a combination of laser-assisted microdissection (LAM), Affymetrix GeneChip profiling (ATH1), and RNA-Seq (SOLiD), we studied the transcriptome of isolated AICs, as well as egg, central and synergid cells from *B. gunnisoniana*. Statistical data analysis revealed the significant enrichment of polyamine and spermidine metabolism in the AIC as compared to the cells of the mature female gametophyte in *Boechera*. In addition, we compared the gene expression profiles of the AIC and the MMC, egg cells and central cells between apomictic *Boechera* and sexual *Arabidopsis*. This uncovered differential expression of genes in important regulatory pathways, including protein degradation, hormonal pathways, cell cycle control, signal transduction, transcriptional regulation, and epigenetic pathways.

## Results

### *Boechera gunnisoniana* seeds are derived from unreduced female gametes

**Figure 1:**
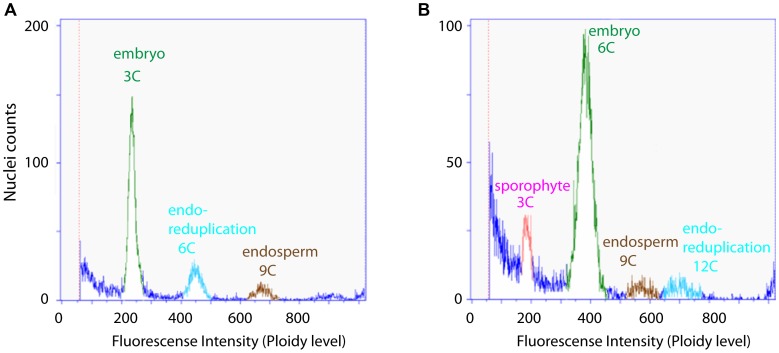
Flow cytometric seed screen on single seeds of *Boechera gunnisoniana* to analyse ploidy. Fluorescence intensity was measured on individual green seeds of *Boechera* to determine the ploidy level of embryo and endosperm. 98% of the seeds measured (N = 84) showed a 3C∶9C ratio of embryo∶endosperm, indicating diplosporous apomixis (A). The remaining 2% showed a 6C∶9C embryo∶endosperm ratio indicative of a BIII hybrid where the embryo is derived from fertilization of an unreduced egg cell (B).

*B. gunnisoniana* has previously been described as diplosporous apomict [48], [49]. While the embryo develops parthenogenetically, the endosperm requires fertilization (pseudogamy) [48], [49]. Based on flow cytometric studies of single seeds, a high variability of the reproductive mode - ranging from obligate sexual to obligate apomictic - has been reported among 71 *Boechera* accessions analysed [50]. We applied this technique to test the frequency of apomictic reproduction in *B. gunnisoniana*. From 84 individual seeds tested, ∼98% showed a 3C∶9C (embryo∶endosperm) ploidy ratio in the seed, as expected for a triploid, pseudogamous apomict (Figure 1A). In two seeds (∼2%) a 6C embryo resulted from fertilization of an unreduced egg cell (Figure 1B). In conclusion, *B. gunnisoniana* reproduces obligatory by pseudogamous apomixis. In all seeds analysed an unreduced egg cell gave rise to the embryo, and embryos developed parthenogenetically at very high frequency.

Nevertheless, the possibility of developmental variations during germline formation cannot be excluded based on a flow cytometric analysis alone. We used ovule and seed clearings for cytological analyses to address the question whether there is potential variation of reproductive development. In young ovules typically a single enlarged subepidermal cell specified to an AIC (Figure S1A, B), while in 3.6% of all ovules (N = 551) an additional enlarged, subepidermal cell was observed (Figure S1A). As previously reported, the AICs give rise to the formation of dyads [48], [49], [51]. Dyad formation was seen at a frequency of 85% (N = 224; Figure S1E, Q). In an additional 10% of all ovules, either dyads accompanied by large parietal cells and or triads were formed (Figure S1F, Q). These two possibilities could not clearly be discriminated based on morphology. Unexpected numbers of nuclei during AIC division or the formation tetrads were observed in ∼2% of all cases (Figure S1G, Q). In the remaining 3% of ovules the AICs apparently failed to divide (Figure S1C, Q), likely leading to developmental arrest (Figure S1D). Formation of a mature gametophyte was observed in 92% of all ovules (N = 353) in agreement with previously published results [49], the majority showing a delay or defect in the fusion of the polar nuclei (Figure S1I, J, R). In 7.4% of the ovules development was arrested early (at AIC or FMS stage), was delayed, or resulted in an unexpected number of nuclei (Figure S1R). At a very low frequency (0.6%) more than one gametophyte developed in a single ovule (Figure S1K, R). In agreement with previous reports, in the absence of pseudogamous fertilization no evidence for the initiation of embryo development was observed [48], [51]. After fertilization, 62% of the seeds developed normally (N = 477; Figure S1L, M). In the remainder, ovules harbouring mature gametophytes or enlarging seeds due to seed coat growth without embryo or endosperm development were observed, or only embryo or endosperm development initiated (Figure S1N–P), suggesting a problem in fertilization. In summary, in *B. gunnisoniana* the large majority of mature gametophytes are formed by diplospory and 98% of the seeds are derived parthenogenetically under our growing conditions. Thus *B. gunnisoniana* is well suited as a model species for molecular studies of apomixis.

### Sequencing, assembly, and annotation of a *Boechera gunnisoniana* reference transcriptome based on ovule tissues

The close relation of the apomict *B. gunnisoniana* with the sexual model species *A. thaliana* provides an excellent basis for comparative analyses. However, while genome sequencing projects for *Boechera* species are currently ongoing (http://www.jgi.doe.gov/), this initiative does not include *B. gunnisoniana*, which is fast cycling and obligatory diplosporous. Thus, as a tool for transcriptomic studies, we generated a reference transcriptome for this species. We isolated ovules at the two developmental stages of interest, megasporogenesis (i.e. ovule stages from the initiation of integument development until the integuments start to overgrow the nucellus; Figure S1A, B) and mature gametophyte stage (Figure S1I–K). The highly enriched ovule samples included some pistil tissue, particularly for the early developmental stage. We prepared two libraries that were sequenced with the Illumina HiSeq2000. Both libraries were assembled together using trinity [52]. Following removal of reads with low average quality scores (Q<30) or adaptor sequences, and trimming of low quality (Q<20) ends, around 697 million reads were assembled into 112'232 sequences corresponding to 30'298 distinct genes with 50% having a sequence length of ≥2'153 bp. The reference transcriptome was annotated using Blast2GO [53] and BLAT [54] (Table S1). Using Blast2GO, 51% of all hits matched best to *A. thaliana* and an additional 25% to *A. lyrata* sequences. Gene ontology (GO) terms could be successfully assigned to 62% of all hits. In addition, we aligned the sequences to cDNA (TAIR10) using BLAT and identified 19'617 close *A. thaliana* homologues of *B. gunnisoniana* genes (hereafter denoted as *Arabidopsis* homologues, Table S2). In summary, the length of assembled sequences and annotation results indicate a good quality of our apomictic reference transcriptome.

### Transcriptional profiling of cells involved in key steps of gametophytic apomixis

**Figure 2:**
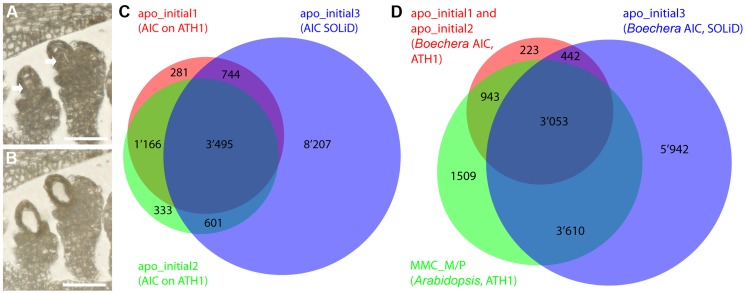
Laser-assisted microdissection (LAM) and transcriptome analysis to study the *Boechera* apomictic initial cell (AIC). (A, B) LAM of the AIC from a 6 µm dry section (scale bar = 40 µm). (A) An ovule harbouring the AIC before LAM. Arrows point to the AICs. (B) The ovule after the AIC has been dissected and collected. (C, D) Venn diagrams showing the overlaps of prediction of expression (P calls; apo_initial1, apo_initial2, MMC_M/P) as determined with the *Bg*PANP algorithm or the *At*PANP algorithm described previously [12], and the genes with ≥5 read counts on *Boechera* homologues (apo_initial3) to the *Arabidopsis* genes as determined by mapping to the *Boechera* reference transcriptome.

For the sexual model plant *Arabidopsis*, transcriptomes of the cell types of the mature gametophyte (egg, central, and synergid cells) and the MMC have been described [11]–[13]. From these studies, important new insights into the transcriptional basis of sexual germline development could be gained. We applied LAM to isolate the AIC and the surrounding sporophytic nucellus tissue, as well as the egg, central, and synergid cells from *B. gunnisoniana* (Figure 2A, B; Figure S2A). For the AIC, small contamination with surrounding nucellus tissue cannot be completely avoided (Figure 2A, B). Due to the dense structure of the mature embryo sac, samples for egg, central, and synergid cells are highly enriched in these cell types, but contain some contamination from neighbouring gametophytic cells (Figure S2A). For transcriptional profiling, 300–650 cell- or tissue-specific sections were pooled per sample. Transcriptional profiling was done using two alternative strategies: heterologous hybridization of amplified and labelled *Boechera* RNA to the Affymetrix ATH1 GeneChip designed for *Arabidopsis* and SOLiD V4 sequencing (Table 1, Figure 2C, D). For GeneChip analysis, the extracted RNA was subjected to linear amplification, labelled and hybridized to the microarray as described [12]. Cross-species hybridization of microarrays with RNA from a species other than the original target species is largely influenced by the degree of sequence similarities between the probes on the array and the mRNA sequence of the species under investigation [55]. To account for this effect we used an adapted *Bg*PANP algorithm for the generation of presence/absence p values, similar to the *At*PANP previously shown to outperform the default algorithm [11]. These algorithms use probes that do not match to the reference genome or transcriptome of the target species as “negative probes” to estimate the true background of each array. For our *Bg*PANP algorithm probes not aligning to the reference transcriptome (allowing for three mismatches) were defined as negative. In this way, several thousand genes were detected significantly above background (hereafter referred to as present/“P”) in each cell type-specific sample (Table 1, Figure 2C, Figure S2B, C, Table S3). For RNA-Seq, the isolated RNA was subjected to linear amplification following an established protocol [13], [56]. Each library was sequenced on one eights of a slide, resulting in 53'701'313 (AIC, apo_initial3), 50'453'327 (egg cell, egg_cell2), 49'331'759 (central cell, central_cell2), and 46'240'916 (synergid cells, synergid_cell2) reads. Reads were processed and aligned to the assembled reference transcriptome as described [13]. Under the applied criteria, between 30% and 37% of the reads had at least one valid alignment, corresponding to 16'371'464 (apo_initial3), 18'783'550 (egg_cell2), 17'348'718 (central_cell2), and 15'353'384 (synergid_cell2) weighted alignments. Gene expression values were calculated as the sum of expression of individual variants (Table S4). We identified 16'385, 17'828, 19'091, and 10'409 *B. gunnisoniana* genes to be expressed (i.e. to have at least 5 mapped reads) in the AIC, egg, central, and synergid cells, respectively (Table 1). This corresponds to 13'047, 13'811, 14'893, and 9'390 expressed (≥5 read counts) *Arabidopsis* homologues in the AIC, egg, central, and synergid cells, respectively (Table 1, Table S2, Table S4). Between ∼2'000 and 6'000 genes were consistently identified in at least two independent cell type-specific samples (Table 1), in agreement with previous observations on the comparability of microarray and RNA-Seq data and the higher sensitivity and genome-wide coverage reached by RNA-Seq [13].

**Table 1:** Transcriptome analysis of 11 samples from apomictic *Boechera* isolated by LAM.

Sample	Genes present on microarray with *Bg*PANP (p value≤0.02)	Number of *B. gunnisoniana* genes with ≥5 read counts	Number of *Arabidopsis* homologues with ≥5 read counts	Expressed in at least 2 samples (column 1 and column 3)
**apo_initial1**	5'595			6'006
**apo_initial2**	5'686			
**apo_initial3**		16'385	13'047	
**sporo_nucellus1**	5'706			4'345
**sporo_nucellus2**	5'236			
**egg_cell1**	5'835			4'490
**egg_cell2**		17'828	13'811	
**central_cell1**	2'973			2'192
**central_cell2**		19'091	14'893	
**synergid_cell1**	4'149			2'472
**synergid_cell2**		10'409	9'390	

Summary of gene expression from *Boechera* germline samples. Samples apo_initial1 and 2, sporo_nucellus1 and 2, egg_cell1, central_cell1, and synergid_cell1 were hybridized on ATH1 microarrays, apo_initial3, egg_cell2, central_cell2, and synergid_cell2 were analysed using RNA-Seq (SOLiD V4) by mapping of reads to the *B. gunnisoniana* reference transcriptome.

### Independent data confirmation shows apomictic initial cell-enriched expression

Apomixis and sexual reproduction are interrelated developmental processes. Therefore, it is likely that the cell type-specific transcriptome profiles are largely overlapping between the sexual and apomictic mode of reproduction. Nevertheless, differences in expression of a subset of genes are expected due to the differences in reproductive mode and species. To compare the cell type-specific transcriptome profiles between *Boechera* and *Arabidopsis*, we used genes designated as P in two (for AIC) or one (for egg and central cell) microarray sample(s), or were identified as an expressed *Arabidopsis* homologue using RNA-Seq (Table 1). For *Arabidopsis* we used the 9'115 genes with evidence of expression in the MMC [12], 12'769 genes expressed in the egg cell (as described in [11], [12] and SOLiD reads aligned to the reference genome of *Arabidopsis thaliana* (TAIR10)), and 14'661 genes expressed in the central cell ([11], [12] and both samples from [13]). Comparing the genes with evidence of expression from *Arabidopsis* and *Boechera* for MMC/AIC, egg and central cells, we found overlapping expression of 7'606, 9'883, and 10'772 genes, respectively (Figure 2C, D; Figure S2B, C).

**Figure 3:**
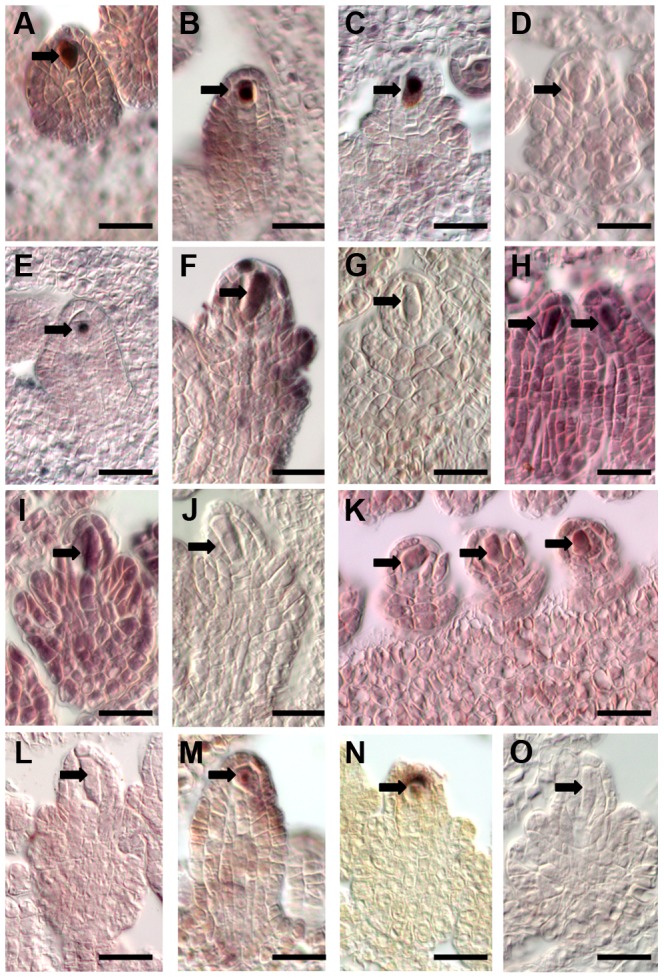
Independent data validation for selected genes by *in situ* hybridization on *B. gunnisoniana* ovules. Data validation for selected genes found expressed in the *Boechera* AIC but not the *Arabidopsis* MMC. Scale bars are 20 µm, arrows point to the AICs. *In situ* hybridizations on *B. gunnisoniana* ovule sections were performed with antisense probes (A–C, E, F, H, I, K, M, N) or sense probes as controls (D, G, J, L, O) for the transcription factors AT1G06170, a basic helix-loop-helix (bHLH) DNA-binding superfamily protein (A–D), AT1G28050, a *B-BOX DOMAIN PROTEIN 13* (E–G), and AT1G76580 a Squamosa promoter-binding protein-like (SBP domain) transcription factor family protein (H–J), an oligopeptide transporter, AT1G59740 (K,L), and AT1G14900, encoding the HIGH MOBILITY GROUP A protein.

In addition, we selected several genes for independent data confirmation by *in situ* hybridization. Based on our analyses, these genes were expressed in the *Boechera* AIC but not in the *Arabidopsis* MMC (Table S5). Probes for *in situ* hybridization on *B. gunnisoniana* ovule sections were designed based on the *Arabidopsis* Col-0 cDNA for three transcription factors (Figure 3, (A–D) AT1G06170, basic helix-loop-helix (bHLH) DNA-binding superfamily protein; (E–G) AT1G28050, *B-BOX DOMAIN PROTEIN 13*; (H–J) AT1G76580, Squamosa promoter-binding protein-like (SBP domain) transcription factor family protein), an oligopeptide transporter (AT1G59740, Figure 3 K,L), and a HIGH MOBILITY GROUP A protein (HMGA, AT1G14900, Figure 3 M–O). The probes were designed to have significant sequence homologies only to the respective *Boechera* homologue (Figure S3, Supporting Information S1). For all selected genes we could confirm enriched expression in the AIC. Taken together, our analyses confirm the accuracy of the *B. gunnisoniana* transcriptome dataset.

### Gene expression and gene ontology enrichment analysis uncovers upregulation of spermidine metabolism in the apomictic initial cell

**Figure 4:**
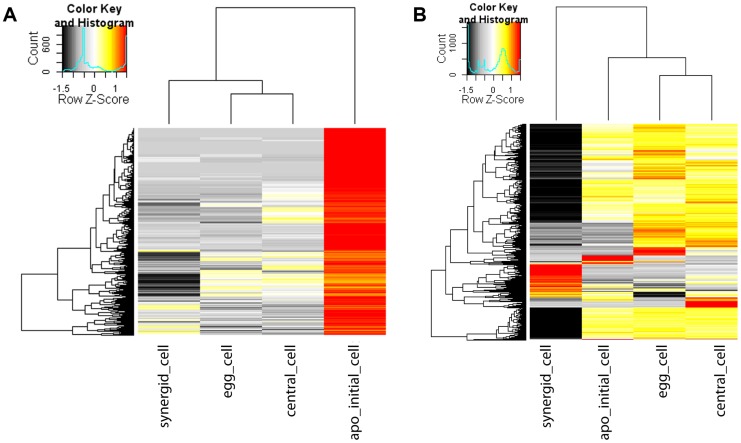
Heatmap of log2 transformed normalized read counts. Heatmap of 1'487 genes enriched in the *Boechera* AIC as compared to all cell types of the mature female gametophyte as identified using NOISeq-sim (A). Heatmap of 3'792 genes enriched in the AIC as compared to all cell types of the mature gametophyte or differentially expressed between any cell type of the mature gametophyte identified using EdgeR (B). The hierarchical clustering of samples and genes was based on euclidean distance and hierarchical agglomerative clustering. Colours are scaled per row and red denotes high expression and black low expression.

Between sexual and apomictic reproduction, there are important differences in cell specification and cell fate decisions. Heterochronic shifts in expression patterns have been reported previously using isolated *Boechera* ovules from sexual and apomictic accessions [27], [28]. However, gene expression has not yet been profiled in a germline-specific manner without the confounding effects of the surrounding sporophytic tissue in *Boechera*. Based on genes significantly enriched in the MMC as compared to the cell types of the mature gametophyte, we previously identified translational control pathways and the activity of RNA-helicases as crucial for the acquisition of germline fate and MMC specification in *Arabidopsis* [12]. To see if similar or different functions are prominent in the *Boechera* AIC as compared to the mature gametophyte, we used read counts obtained by mapping to the *Boechera* reference transcriptome. To identify genes significantly enriched we used NOIseq-sim, a non-parametric approach for differential expression analysis based on simulated replicate samples [57]. We identified 1'487 genes to be significantly enriched in the AIC as compared to the cell types of the mature gametophyte (Figure 4A). In addition, 3'509, 1'466, and 1'806 genes were significantly enriched in the egg, central, and synergid cells, respectively, as compared to the other three cell types of the germline under investigation.

In a gene ontology (GO) analysis, we identified functions important for pollen germination and sperm cell and pollen maturation as significantly enriched in the AIC (p<0.01, Table 2). In addition, different metabolic and transport processes were upregulated, in addition to spermidine metabolism and polyamine biosynthesis (p<0.01, Table 2). Functions related to plant cell wall modification and epigenetic regulatory pathways (histone H3K4 demethylation and maintenance of DNA methylation) were also amongst the enriched functions (p<0.01, Table 2). Furthermore, cytokinin catabolism was among the near-significantly enriched processes (p = 0.012, Table 2).

**Table 2:** Gene ontology analysis.

GO.ID	Term	Annotated	Significant	Expected	p value
GO:0010584	pollen exine formation	92	44	4.73	<1e-30
GO:0009827	plant-type cell wall modification	336	46	17.26	1.20E-09
GO:0008216	spermidine metabolic process	25	10	1.28	2.00E-07
GO:0009860	pollen tube growth	393	43	20.19	2.50E-06
GO:0006527	arginine catabolic process	8	5	0.41	1.70E-05
GO:0006817	phosphate ion transport	32	9	1.64	2.30E-05
GO:0008643	carbohydrate transport	97	18	4.98	2.30E-05
GO:0046467	membrane lipid biosynthetic process	210	25	10.79	8.00E-05
GO:0006596	polyamine biosynthetic process	24	7	1.23	0.00015
GO:0016036	cellular response to phosphate starvation	194	22	9.97	0.00042
GO:0055085	transmembrane transport	1020	80	52.4	0.00048
GO:0034720	histone H3-K4 demethylation	5	3	0.26	0.00125
GO:0009396	folic acid-containing compound biosynthetic process	18	5	0.92	0.00173
GO:0030162	regulation of proteolysis	6	3	0.31	0.0024
GO:0048235	pollen sperm cell differentiation	47	8	2.41	0.00248
GO:0046938	phytochelatin biosynthetic process	7	3	0.36	0.00405
GO:0006665	sphingolipid metabolic process	88	12	4.52	0.00582
GO:0030036	actin cytoskeleton organization	254	14	13.05	0.00587
GO:0010216	maintenance of DNA methylation	15	4	0.77	0.00599
GO:0009395	phospholipid catabolic process	15	4	0.77	0.00599
GO:0010951	negative regulation of endopeptidase activity	8	3	0.41	0.00623
GO:0010199	organ boundary specification between lateral organs and meristems	8	3	0.41	0.00623
GO:0015800	acidic amino acid transport	7	3	0.36	0.00763
GO:0010084	specification of organ axis polarity	3	2	0.15	0.00764
GO:0090408	phloem nitrate loading	3	2	0.15	0.00764
GO:0042398	cellular modified amino acid biosynthetic process	84	10	4.32	0.01076
GO:0010205	photoinhibition	18	4	0.92	0.01188
GO:0009823	cytokinin catabolic process	10	3	0.51	0.01236

Biological Processes identified to be up-regulated based on 1'487 genes identified to be up-regulated in the AIC as compared to the cell types composing the mature gametophyte (egg cell, central cell, synergid cells).

In the egg cell of *Boechera*, cytokinin metabolism is a dominant molecular function as discovered by analysis of GO enrichment based on the 3'509 significantly upregulated genes, in addition to transcription factor activity (Table S6A). The central cell transcriptome is dominated by different epigenetic regulatory pathways, cell cycle regulation, and regulation of cell fate decisions (p<0.01, Table S6B).

At higher stringency, using EdgeR with an estimated biological variation coefficient of 0.8, we identified 142 genes to be significantly enriched in all pairwise comparisons of the AIC with the transcriptomes of cells of the mature gametophyte (adjusted p value (FDR)<0.05, Benjamini-Hochberg adjustment; Figure 4B) [58]. Based in these genes, GO enrichment analysis confirmed spermidine metabolism, cytokinin catabolism, and functions related to pollen development and germination as significantly enriched in the AIC (p<0.01; Table S7). Notably, also the term “sexual reproduction” was an enriched function based on upregulated genes. In addition, 3'792 genes were differentially expressed in any pairwise comparison between the cell types of the mature gametophyte (FDR≤0.05 for comparisons between synergid cells and egg- or central cell, or an unadjusted p value≤0.001 for comparisons between egg cell and central cell (Figure 4B)).

In summary this indicates interesting differences in the functions underlying the specification of the germline lineage and the female gametes in the apomict *B. gunnisoniana* as compared to the sexual pathway in *Arabidopsis*. Consistently, spermidine metabolism was identified as enriched in the AIC. Our analysis also indicated a distinct regulation of cytokinin metabolism and degradation in the apomictic germline lineage.

### Evidence for different regulation of important regulatory pathways in apomictic and sexual germline cells

To analyse differences in gene activity between the sexual and apomictic germline in more detail, we identified *Arabidopsis* genes and their homologues only expressed in a certain cell type in *Arabidopsis* or *Boechera*. *Boechera* genes were designated as expressed when having at least 5 read counts by mapping against the reference transcriptome, or a P call on one or both microarrays. For a conservative estimate of genes only expressed in *Arabidopsis*, we also aligned the SOLiD reads to the reference genome of *A. thaliana* (TAIR10) and only considered genes with at least 5 read counts. We included the latter method as annotation of the closest *Arabidopsis* homologue is not always unambiguous. Sometimes sequence variants for one *Boechera* gene have their highest sequence similarity to different *Arabidopsis* genes (see below), complicating a direct comparison. Of the 9'115 MMC-expressed genes, no evidence of expression has been found for 852 genes in the AIC. GO analysis on this set of genes identified a significant enrichment of different molecular functions, including metabolism, regulation of physiological responses, auxin turnover, translation initiation, and functions related to cell wall structure and cell cycle control (p<0.01, Table 3A). Also the “core cell cycle genes” were found to be significantly enriched (Fisher's exact test, p = 0.006), in agreement with the meiotic fate of the MMC. In addition, 14 protein family (PFAM) domains were identified as enriched (Fisher's exact test, p value<0.01, Table S8) including F-box domain and F-box related domains, as well as the cyclin C- and N-terminal domains. This suggests that protein ubiquitinylation and degradation, as well as cell cycle control, may be differentially regulated between MMCs and AICs. Using a similar approach, out of 12'679 genes expressed in the *Arabidopsis* egg cell (Figure S2B) we identified 1'731 for which no homologues were expressed in the *Boechera* egg cell. GO analysis in this set of genes identified biological processes related to RNA modification and splicing, transport and metabolism, and methylation-dependent chromatin silencing as significantly enriched, and also functions related to double fertilization and endosperm formation (p<0.01, Table 4A). In addition, two transcription factor families, the “*At*RKD Transcription Factor Family” and the “MYB Transcription Factor Family” were identified as significantly enriched gene families (Fisher's exact test, p = 0.0087 and p = 0.0038, respectively). For the *Arabidopsis* central cell, out of 14'661 expressed genes (Figure S2C) no evidence for expression of homologues in the *Boechera* central cell was found for 2'146 genes. As in the *Arabidopsis* egg cell, biological processes related to RNA modification and splicing (GO:0000154, rRNA modification, p = 5.1e-17; GO:0045292, nuclear mRNA *cis*-splicing, via splicosome, p = 5.4e-5) and “endosperm development” (p = 0.0078) were significantly enriched. In addition, out of the 12 PFAM domains identified as enriched, three were related to F-box domains (Fisher's exact test, p<0.01, Table S9).

**Table 3:** Gene ontology analysis on MMC and AIC.

A) *Arabidopsis thaliana* MMC					
GO.ID	Term	Annotated	Significant	Expected	p value
GO:0016538	cyclin-dependent protein kinase regulator activity	29	6	1.14	0.00081
GO:0005199	structural constituent of cell wall	30	6	1.18	0.00098
GO:0016844	strictosidine synthase activity	14	4	0.55	0.00176
GO:0004129	cytochrome-c oxidase activity	15	4	0.59	0.00232
GO:0010178	IAA-amino acid conjugate hydrolase activity	3	2	0.12	0.00455
GO:0003743	translation initiation factor activity	82	9	3.24	0.00496
GO:0005034	osmosensor activity	4	2	0.16	0.00885

B) *Boechera gunnisoniana* AIC					
GO.ID	Term	Annotated	Significant	Expected	p value
GO:0005275	amine transmembrane transporter activity	69	14	2.94	1.40E-05
GO:0022843	voltage-gated cation channel activity	28	6	1.19	0.00098
GO:0050662	coenzyme binding	321	27	13.66	0.00121
GO:0072547	tricoumaroylspermidine meta-hydroxylase activity	2	2	0.09	0.00181
GO:0072548	dicoumaroyl monocaffeoyl spermidine meta-hydroxylase activity	2	2	0.09	0.00181
GO:0072549	monocoumaroyl dicaffeoyl spermidine meta-hydroxylase activity	2	2	0.09	0.00181
GO:0016620	oxidoreductase activity, acting on the aldehyde or oxo group of donors	42	7	1.79	0.00181
GO:0005315	inorganic phosphate transmembrane transport activity	14	4	0.6	0.00232
GO:0016844	strictosidine synthase activity	14	4	0.6	0.00232
GO:0004091	carboxylesterase activity	109	12	4.64	0.00232
GO:0016160	amylase activity	17	4	0.72	0.00498
GO:0000257	nitrilase activity	3	2	0.13	0.00528
GO:0016298	lipase activity	107	11	4.55	0.00586
GO:0004708	MAP kinase kinase activity	11	3	0.47	0.00981

Significant upregulation of molecular functions based on 852 genes expressed in the *Arabidopsis* MMC but not in the *Boechera* AIC (A). Significant upregulation of molecular functions based on 901 genes expressed in the apomictic initial cell but not in the MMC (B). A p value<0.01 was considered significant.

**Table 4:** Gene ontology analysis on sexual and parthenogenetic egg cells of *Arabidopsis* and *Boechera*, respectively.

(A) *Arabidopsis thaliana* egg cell					
GO.ID	Term	Annotated	Significant	Expected	p value
GO:0000154	rRNA modification	74	43	4.02	<1e-30
GO:0045292	nuclear mRNA cis splicing, via spliceosome	8	4	0.43	0.00051
GO:0045490	pectin catabolic process	2	2	0.11	0.00295
GO:0015986	ATP synthesis coupled proton transport	38	7	2.07	0.00395
GO:0043086	negative regulation of catalytic activity	82	11	4.46	0.00474
GO:0019432	triglyceride biosynthetic process	7	3	0.38	0.00475
GO:0080155	regulation of double fertilization forming a zygote and endosperm	3	2	0.16	0.00854
GO:2000014	regulation of endosperm development	3	2	0.16	0.00854
GO:0090309	positive regulation of methylation-dependent chromatin silencing	3	2	0.16	0.00854

(B) *Boechera gunnisoniana* egg cell					
GO.ID	Term	Annotated	Significant	Expected	p value
GO:0006468	protein phosphorylation	1135	308	199.51	2.70E-15
GO:0006355	regulation of transcription, DNA-dependent	1868	421	328.36	2.20E-08
GO:0007169	transmembrane receptor protein tyrosine kinase signalling pathway	130	49	22.85	4.00E-08
GO:0007623	circadian rhythm	100	33	17.58	0.00014
GO:0006952	defense response	884	201	155.39	0.00016
GO:0009739	response to gibberellin stimulus	123	42	21.62	0.00043
GO:0009641	shade avoidance	13	8	2.29	0.0005
GO:0019747	regulation of isoprenoid metabolic process	10	7	1.76	0.00059
GO:0007165	signal transduction	1319	314	231.85	0.00067
GO:0006974	response to DNA damage stimulus	228	61	40.08	0.00068
GO:0006261	DNA-dependent DNA replication	65	23	11.43	0.00092
GO:0010321	regulation of vegetative phase change	4	4	0.7	0.00095
GO:0010215	cellulose microfibril organization	4	4	0.7	0.00095
GO:0000079	regulation of cyclin-dependent protein serine/threonine kinase activity	46	17	8.09	0.00139
GO:0009314	response to radiation	570	131	100.19	0.00209
GO:0010158	abaxial cell fate specification	7	5	1.23	0.00257
GO:0009737	response to abscisic acid stimulus	412	95	72.42	0.0026
GO:0016998	cell wall macromolecule catabolic process	30	12	5.27	0.00321
GO:2000038	regulation of stomatal complex development	10	6	1.76	0.00323
GO:0010065	primary meristem tissue development	10	6	1.76	0.00323
GO:0006571	tyrosine biosynthetic process	5	4	0.88	0.0041
GO:0009939	positive regulation of gibberellic acid mediated signalling pathway	5	4	0.88	0.0041
GO:0046482	para-aminobenzoic acid metabolic process	5	4	0.88	0.0041
GO:0042372	phylloquinone biosynthetic process	8	5	1.41	0.00585
GO:0010162	seed dormancy process	21	9	3.69	0.0061
GO:0010200	response to chitin	127	34	22.32	0.0063
GO:0009826	unidimensional cell growth	211	52	37.09	0.00764
GO:0006928	cellular component movement	93	26	16.35	0.00871
GO:0016556	mRNA modification	15	7	2.64	0.00889
GO:0048868	pollen tube development	116	31	20.39	0.00903
GO:0009694	jasmonic acid metabolic process	29	11	5.1	0.00957
GO:0006499	N-terminal protein myristoylation	431	95	75.76	0.00974
GO:0006857	oligopeptide transport	76	22	13.36	0.00986

Significant enrichment of biological processes based on 1'731 genes with evidence of expression only in the sexual *Arabidopsis* egg cell (A) and 5'273 *Boechera* homologues with evidence of expression only in the parthenogenetic egg cell (B). A p value<0.01 was considered significant.

For the identification of genes only expressed in the apomictic *Boechera* germline and not in *Arabidopsis*, we used the *Arabidopsis* homologues identified and mapping to the *Boechera* reference transcriptome, combined with the microarray data. We identified 5'273 and 4'902 genes expressed in the apomictic egg and central cell, respectively, that were absent in the corresponding *Arabidopsis* cell type. We used more restrictive criteria to identify the 901 genes expressed in the AIC but not in the MMC: we considered only *Arabidopsis* homologues with ≥5 reads in the SOLiD dataset and detected as P in at least one microarray dataset of the AIC. Interestingly, for all three cell types of the apomictic *Boechera* germline, GO and/or PFAM analyses revealed a significant enrichment of signal transduction processes and protein kinases (Table 3B, Table 4B, Table 5, Table S10). For instance, we identified the significant enrichment of “MAP kinase kinase activity” in the AIC (p<0.01, Table 3B). In addition, transport and metabolic processes were enriched, and spermidine metabolism was confirmed as an important feature (p<0.01, Table 3B). Analysis of gene families revealed the Squamosa promoter Binding Proteins as enriched (Fisher's exact test, p value<0.01, SBP transcription factor family). Analysis of gene families and PFAM domains also identified a significant enrichment of the *AMINO ACID/AUXIN PERMEASE* (*AAAP*) family, the *ARF* transcription factor family, and the protein domain of the AUX/IAA family during apomictic germline specification (Fisher's exact test, p<0.01, Table 6). Also the family of B3 transcription factors (B3_TFs), including the *ARF* transcription factor family, was identified as significantly enriched (Fisher's exact test, p<0.01, Table 6). In the parthenogenetic *Boechera* egg cell, GO analysis suggests the importance of signal transduction pathways, cell cycle regulation, and transcription factor activity (p<0.01). In general, in the female gametes analysis of gene families identified the enriched expression of several transcription factor families, particularly the basic Helix-Loop-Helix transcription factors both in the egg and central cell (Table S11).

**Table 5:** Analysis of PFAM domains significantly enriched in the *Boechera* germline.

AIC				
ID	Significant	Expected	p value	description
PF01535	217	124.07	2.30E-10	Pentatricopeptide repeat
PF00069	268	200.47	0.00012525	Protein kinase domain
PF00225	38	18.94	0.00123647	Kinesin motor domain
PF00612	32	16.10	0.00295969	IQ calmodulin-binding motif
PF07714	130	96.92	0.00671958	Protein typrosine kinase

egg cell				
ID	Significant	Expected	p value	description
PF00010	42	24.88	0.00476216	Helix-loop-helix DNA-binding domain
PF00069	183	131.90	0.00016034	Protein kinase domain
PF00560	131	73.15	6.30E-08	Leucine rich repeat
PF00730	9	2.25	0.00288164	HhH-GPD superfamily base excision DNA repair protein
PF00786	10	2.99	0.004385	P21-Rho binding domain
PF00931	68	31.43	5.85E-07	NB-ARC domain
PF01535	197	82.32	3.67E-21	Pentacortico repeat
PF01582	42	24.88	0.00476216	Toll Interleukin receptor
PF07646	12	3.74	0.00238746	Kelch motif
PF07714	100	63.80	0.00017157	Protein tyrosine kinase
PF07725	34	15.34	0.00027842	Leucine rich repeat, LRR_3
PF08263	75	39.10	5.75E-06	Leucine rich repeat N-terminal domain

central cell				
ID	Significant	Expected	p value	description
PF00010	48	26.1526398	0.00058139	Helix-loop-helix DNA-binding domain
PF00069	222	138.628655	7.03E-09	Protein kinase domain
PF00076	23	48.1759154	0.00025011	RNA recognition motiv
PF00225	26	11.9948198	0.00198709	Kinesin motor domain
PF00560	137	76.8848283	4.57E-08	Leucine rich repeat
PF00612	24	11.0116378	0.00227686	IQ calmodolin binding domain
PF00931	64	33.0349134	2.48E-05	NB-ARC domain
PF01486	19	8.06209197	0.00435678	K-box domain
PF01535	191	86.5200114	1.47E-17	Pentacorticopeptide repeat
PF01582	44	26.1526398	0.00422265	Toll-interleukin receptor
PF02183	12	4.12936418	0.00722254	Homeobox associated leucine zipper
PF02362	33	18.2871842	0.00530936	B3 domain
PF02458	24	12.3880925	0.00850195	Transferase family
PF03000	16	6.48900085	0.00559973	NPH3-family
PF03514	17	6.48900085	0.00323279	GRAS domain family
PF04570	11	3.14618223	0.00233681	DUF581
PF04770	13	3.34281862	0.00047255	ZF-HD protein dimerisation region
PF04784	10	3.14618223	0.0059545	DUF547
PF07714	115	67.0530088	2.29E-06	Tyrosine kinase
PF07725	36	16.1241839	0.00015028	Leucine rich repeat, LRR3
PF08263	84	41.0970054	2.01E-07	Leucine rich repeat N-terminal domain
PF11721	16	6.29236446	0.005044	Di-glucose binding with endoplasmatic reticulum

PFAM domains significantly enriched in 901, 5'273 and 4'902 genes expressed only in the AIC, egg cell and central cell of *Boechera* but without evidence of expression in the *Arabidopsis* MMC, egg cell, and central cell, respectively (p value<0.01).

**Table 6:** Gene family enrichment.

Gene family	Significant	Expected	p value
AAAP family	8	1.55	0.0004025
Acyl Lipid Metabolism Family	35	20.65	0.0050592
ARF Transcription Factor Family	5	0.71	0.0015891
B3_TFs	8	2.77	0.0099967
Glycoside Hydrolase Gene Families	24	13.30	0.0084674
Monolignol Biosynthesis	8	2.31	0.0037947
Organic Solute Cotransporters	28	10.20	7.53E-06
SBP Transcription Factor Family	4	0.59	0.0051551
Superfamily of zinc-coordinating DNA-binding proteins	4	0.59	0.0051551

Enrichment of gene families in 901 genes with evidence of expression in the AIC but not in the sexual MMC as analysed by Fisher's exact test. A p value<0.01 was considered significant.

In summary, our analysis reveals interesting differences in the regulatory programs underlying the acquisition of germline fate and between the female gametes. While the subset of genes only expressed in the sexual germline is significantly enriched in protein degradation pathways, the apomictic *Boechera* germline is marked by the activity of signal transduction processes. In addition, indications for a role of auxin signalling and metabolism were observed in both germlines. Among the genes identified as active in the apomictic germline lineage only, we found the enrichment of different transcription factors families, particularly basic helix-loop-helix transcription factors in the female *Boechera* gametes. The comparison between the sexual and apomictic germlines further revealed differential regulation of genes involved in cell cycle control and posttranscriptional regulatory processes, including mRNA splicing, and epigenetic regulatory pathways related to methylation-dependent chromatin modifications.

### Expression analysis of selected candidate genes and pathways related to apomixis

For a number of genes, enriched expression in the *Arabidopsis* MMC or the aposporic initial cell of *Hieracium praealtum* have previously been described [12], [31]. In addition, for sexual or apomictic germline development, evidence for the importance of different genes including core cell cycle genes, meiotic genes, and genes involved in epigenetic regulatory pathways has previously been reported based on mutant analyses or expression patterns [42], [43], [59]. Thus, we compared the expression of selected genes of interest upon sexual and diplosporic germline initiation.

From a list of 89 core cell cycle genes as defined before [60], [61], 75 are represented on the ATH1 array and for 66 *Arabidopsis* genes, homologues were identified in the *Boechera* reference transcriptome. From these, 41 genes are expressed in the *Arabidopsis* MMC and 49 homologues are present in the *Boechera* AIC. 16 and 24 cell cycle regulators have only been detected in the MMC or the AIC, respectively (Table S12). In particular, the genes only detected in the apomict upon germline specification include genes involved in different cell cycle transitions, e.g. G1/S phase, including a number of genes involved in the cyclin D/retinoblastoma/E2F pathway (Table S12). The observed differences in cell cycle regulation are in agreement with the different mechanisms of cell division in the meiotic MMC *versus* the diplosporous AIC.

**Figure 5:**
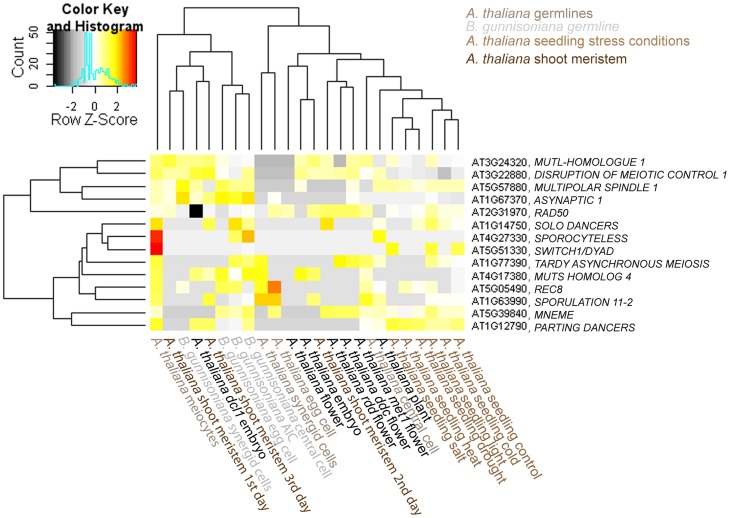
Heatmap of log2 transformed normalized read counts for 14 selected meiotic or MMC-expressed genes. Hierachical clustering of read counts from different *Arabidopsis* and *Boechera* cell- and tissue types [13], [62]–[66]. The hierarchical clustering of samples and genes was based on euclidean distance and hierarchical agglomerative clustering. Colours are scaled per row. Red denotes high expression and black low expression.

Interestingly, for 14 selected meiotic genes and genes expressed in the sexual MMC no evidence for expression was found in the *H. praealtum* aposporous initial cell [31]. However, although the aposporous and the diplosporous initial cell both give rise to unreduced embryo sacs, cell lineage and developmental fate are markedly different. So far it is unknown whether common regulators underlie apomeiosis in these distinct types of apomixis. Interestingly, for all 14 genes except for *SWITCH*/*DYAD* and *SPO11-2* evidence for expression was found in the *Boechera* AIC; although at very low levels for most genes (Figure 5). The *Arabidopsis* male meiocytes cluster separately from the expression data of different *Arabidopsis* cell- and tissue-types publicly available [13], [62]–[66]. Furthermore, the RNA helicase *MEM* previously identified as predominantly expressed in the *Arabidopsis* MMC is only expressed at low levels in the AIC but higher in the apomictic egg cell (Figure 5). Interestingly, this indicates differences in the expression of genes previously identified to have important functions for MMC specification and meiosis in *Arabidopsis*. In agreement with the differences in developmental fate, the data also suggest differences in cell specification of aposporous and diplosporous initial cells. Nevertheless, the majority of 35 genes described as enriched in the *H. praealtum* aposporous initial cell or the early apomictic embryo sac as compared to sporophytic ovule tissues [31] was also expressed in the *Boechera* AIC, except for *HISTONE ACETYLTRANSFERASE OF THE CBP FAMILY1*, *LIKE HETEROCHROMATIN PROTEIN1*, *BEL1-LIKE HOMEODOMAIN1*, *CONSTITUTIVE DISEASE RESISTANCE1*, genes involved in lipid localization (*AT1G03103*, *AT5G38170*, *AT3G18280*, *AT1G43666*), and a pathogenesis-related lipid-transfer protein gene (*AT2G18370*).

Increasing evidence suggests the involvement of epigenetic regulatory pathways in the discrimination between sexual reproduction and apomixis. Therefore, we were interested in a closer investigation of the expression of 69 genes involved in DNA methylation and small RNA pathways (as used in [12]). 58 of these genes have annotated homologues in *Boechera* (Table S1). 40 genes are consistently present both in the AIC and in the MMC, supporting the important role of epigenetic regulatory pathways for the initiation of germline development [12]. Heatmap clustering suggests the closest relation between the AIC dataset and the datasets of the *Boechera* female gametes (Figure S4). Together, these datasets cluster with the *Arabidopsis* egg and synergid cells, but distantly from male meiocytes or the central cell of the sexual germline lineage (Figure S4). Nevertheless, a number of genes were only detected in the MMC or the AIC, respectively (Supporting Information S2). Genes only detected in the AIC included *ENHANCED SILENCING PHENOTYPE3* (*ESP3*). Also *AGO9* and *RDR6*, mutations in which cause an apospory-like behaviour in *Arabidopsis* [42], were both detected at low levels in the *Boechera* AIC (Figure S4, Figure S5). In summary, for a subset of genes involved in DNA methylation and small RNA pathways, we observed distinct expression patterns during germline specification in sexual *Arabidopsis* MMCs *versus* apomictic *Boechera* AICs, which may be of importance to distinguish fate decisions between these alternative reproductive modes.

### Influence of sequence similarities between *Arabidopsis* and *Boechera* homologues on the distribution of count data

**Figure 6:**
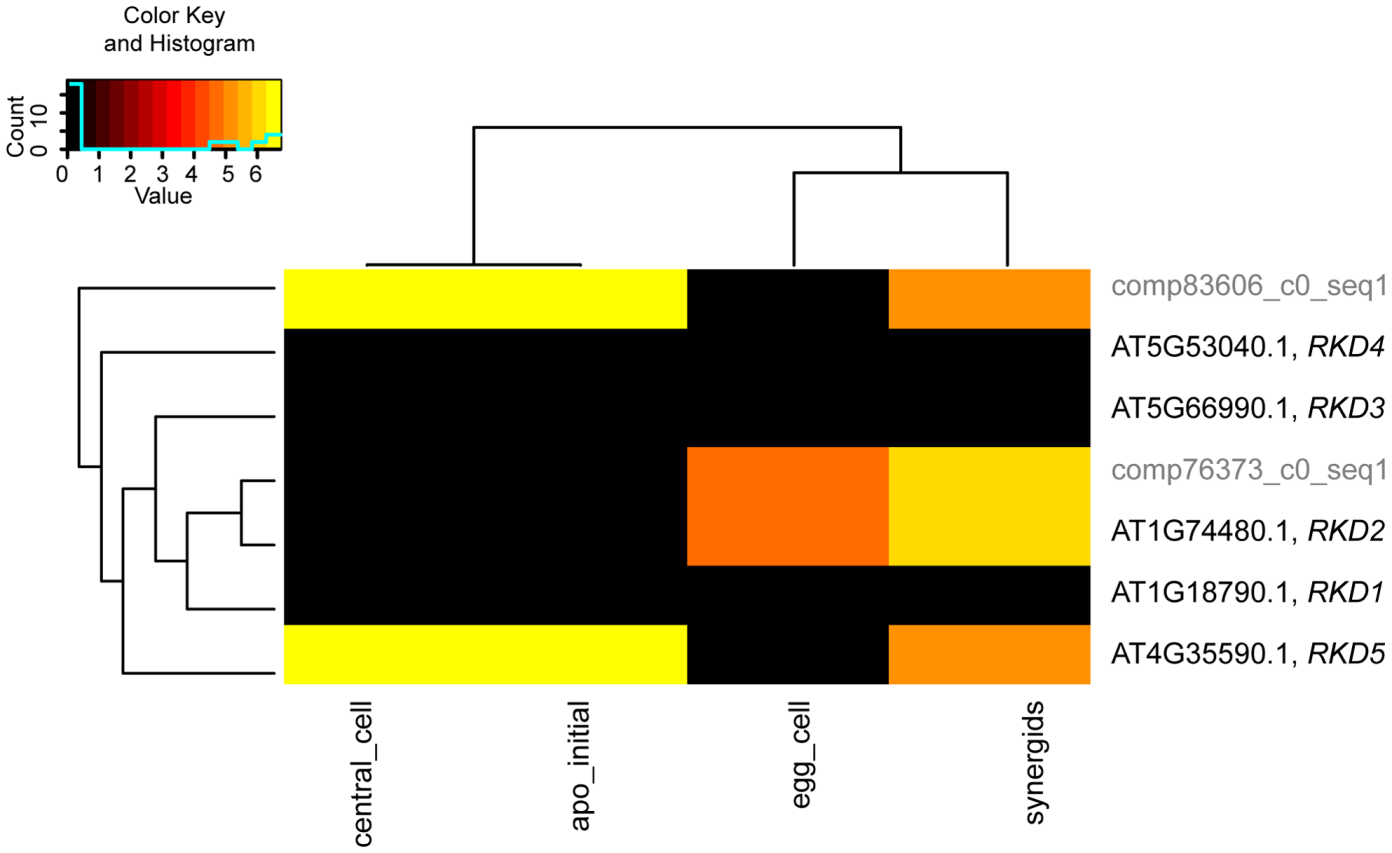
Analysis of sequence divergence of members of the *AtRKD* gene family. Analysis of sequence divergence of members of the *AtRKD* gene family and close *Boechera* homologues as analysed with ClustalX based on protein sequences and read counts assigned. *Boechera* gene model variants are indicated with compxxx_cY_seqZ.

**Figure 7:**
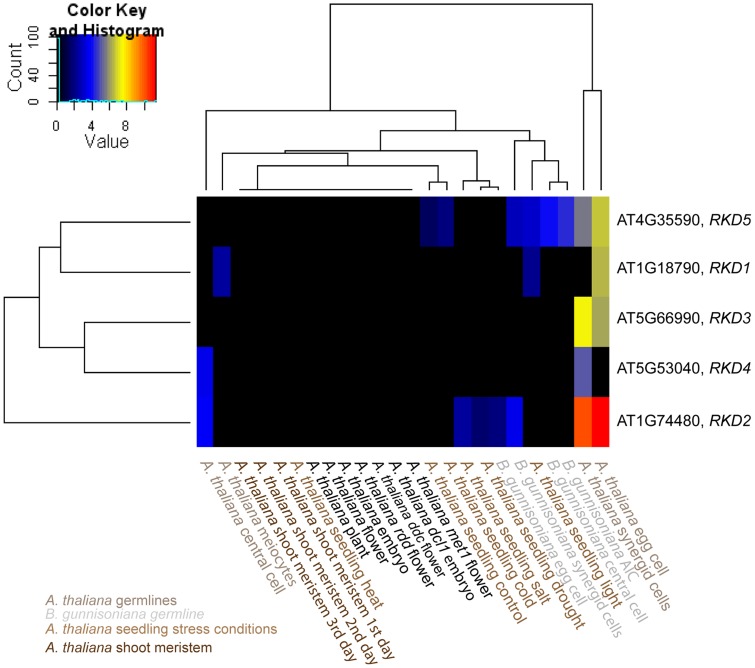
Heatmap clustering of members of the *AtRKD* gene family. Heatmap of normalized log2 transformed read counts from different *Arabidopsis* and *Boechera* cell- and tissue-types [13], [62]–[66]. The hierarchical clustering of samples and genes was based on euclidean distance and hierarchical agglomerative clustering. No row scaling of colours was applied. Red denotes high expression and black low expression.

Particularly within a gene family, the assignment of the closest *Boechera* homologues to *Arabidopsis* genes is not always unambiguous. For selected gene families of interest we aimed to test the influence of sequence divergence and annotation criteria on the expression estimates for *B. gunnisoniana* homologues of *A. thaliana* genes. Identification of the closest homologues in the *Boechera* reference transcriptome was based on the highest bit score sum with BLAT, using only the best mappings per *Arabidopsis* gene. For this analysis we selected the *AtRKD* gene family (Figure 6, 7). In addition, similar analysis of the *ARIADNE* (*ARI*) gene family, and the *AGO* gene family are shown in “Supporting Information S2” (Figure S5, Figure S6, Figure S7, Supporting Information S2).

The *RKD* gene family has been identified in our analysis to be enriched among the genes expressed only in the *Arabidopsis* but not the *Boechera* egg cell. Instead of the five members of the *Arabidopsis RKD* family, two gene models of homologues with one variant each have been identified in the *Boechera* reference transcriptome (Figure 6). This suggests either that the gene family is smaller in *Boechera* as compared to *Arabidopsis*, or that additional members of this family are not expressed in *Boechera* ovules at the developmental stages used to generate the reference transcriptome. Analysis of sequence similarities indicates the closest similarity between comp76373_c0_seq1 and *AtRKD2*. In agreement, counts for reads mapped to comp76373_c0_seq1 are assigned to *AtRKD2*. However, while clustering of comp83606_c0_seq1 indicates higher sequence divergence from all *AtRKDs*, the reads are assigned to *AtRKD5*. The expression and role of members of the *RKD* family in *Arabidopsis*, where they play a role in egg cell specification, has been described previously [67]. As the two *Boechera* gene models homologous to the *AtRKD* genes are expressed in the egg apparatus (egg and synergid cells; Figure 6, 7), the *Arabidopsis* family as a whole is predominantly expressed in the *Arabidopsis* egg apparatus (Figure 7), in agreement with our gene set enrichment analysis.

## Discussion

### *Boechera gunnisoniana* as a model species to study apomixis

To investigate apomictic reproduction, the female germline is in particular of interest, as in apomicts clonal offspring genetically identical to the mother plant is generated. In *B. gunnisoniana*, based on a flow cytometric seed screen using single seeds, we observed exclusively apomeiotic behaviour and only a very low percentage of fertilized, unreduced egg cells. In agreement, the formation of dyads and mature *Polygonum* type embryo sacs were observed at high frequencies. At low frequencies, developmental variations during germline development were observed, including the formation of more than one female gametophyte per ovule. This could either be due to a failure of degradation of the second megaspore resulting from diplospory, or indicate the rare occurrence of apospory. Interestingly, parthenogenesis remains repressed in the absence of pseudogamous fertilization. In maturing siliques, likely due to a lack of successful fertilization, not all female gametes give rise to an embryo or endosperm. As a consequence of deviations from apomictic germline development and fertilization, reproductive development seems to arrests, so that the vast majority of mature seeds are derived apomictically. This obligate apomictic behaviour, together with its fast cycling (about 4 months from seed to seed) and the close relation to the sexual model species *A. thaliana*, make *B. gunnisoniana* an ideal system to study apomixis. We generated the first comprehensive, annotated reference transcriptome for reproductive development in *B. gunnisoniana*, including the identification of *Arabidopsis* homologues, as an essential tool for further studies.

### Spermidine and polyamine metabolism are enriched in the apomictic initial cell

Previously, similarities of germline development were reported even across kingdoms, between the plant and animal germline. These are likely of general importance for the acquisition of germline fate [12]. Nevertheless, cell type specification and developmental fate is markedly different during germline specification in sexual, aposporous, and diplosporous species. Consistently, a number of differences in gene expression profiles have been observed between the apomictic and the sexual germline. In the *B. gunnisoniana* AIC, a number of functions related to pollen development and germination were enriched, consistent with gene activities observed during germline development in apomeiotic, non-parthenogenetic hybrids of *Pennisetum glaucum* [33].

Polyamine biosynthesis and spermidine metabolism were also identified as features of the *Boechera* AIC. Interestingly, spermidine synthesis is essential for embryo development in *Arabidopsis* [68]. In addition, a possible role of polyamines in promoting somatic plant embryogenesis was reported [69]–[71]. This indicates the importance of spermidine for plant reproduction and provides an interesting link between polyamines and somatic embryogenesis, a form of asexual reproduction different from gametophytic apomixis. Interestingly, spermidine is involved in the protection of DNA from oxidative stress by quenching free radicals mostly arising from reactive oxygen species (ROS) [72]. In line with the high activity of spermidine metabolism in the apomeiotic AIC, it has been hypothesized that repair of DNA damage after oxidative stress has been a major driving force for the evolution of meiosis [73]. Apart from being cytotoxic, the role of ROS in signalling and for plant reproductive development has recently been demonstrated [74]. Notably, a spermine/spermidine synthase has previously been identified to be present in the apospory-specific region of *P. squamulatum* and hypothesized to be expressed [75], supporting a potential role of these substances for the specification of the apomictic germline. However, further studies will be required to conclude which, if any, role polyamine and spermidine metabolism plays during germline development or the determination of the asexual reproductive fate.

### Differentially regulated genes and pathways during sexual and apomictic reproduction include hormonal and protein degradation pathways and transcription factor activity

In addition to polyamine and spermidine metabolism, the activities of important hormonal pathways were also observed in the AIC. Upregulation of cytokinin degradation was detected upon apomictic germline specification as compared to the mature gametophyte, while the egg cell is marked by gene activities leading to cytokinin modifications. In addition, genes involved in auxin signalling were enriched in the set of genes expressed in the AIC but not in the sexual MMC, in line with the identification of genes involved in auxin signal transduction in the *H. praealtum* apospory initial cell [31]. In the *Boechera* AIC, we detected an enriched activity of the AUX/IAA and the ARF transcription factor gene families. These play crucial roles in auxin-regulated gene expression, for example to control cell type-specific auxin responses during *Arabidopsis* embryo development [76], [77]. Evidence for differential expression of ARF genes has previously been reported during early stages of reproductive development in a comparative cDNA-AFLP analysis targeting sexual and apomictic *Paspalum simplex* flowers [35].

In contrast, genes active only during sexual reproduction and MMC specification are marked by an enrichment of F-box proteins. F-box proteins play important roles in ubiquitin-dependent protein degradation involved in signal transduction pathways, cell cycle control, and a variety of other processes [78], [79]. The expression of miRNAs targeting genes encoding F-box proteins and ARF transcription factors in *Boechera* floral tissues supports the importance of these pathways in plant reproductive development [80]. This is in line with the identification of a truncated *ARI* allele with homology to *Arabidopsis ARI7* as a candidate apospory locus in *Hypericum perforatum* [81]. *ARI7* encodes a ring finger protein predicted to be involved in ubiquitin-dependent protein degradation [82]. Interestingly, we found evidence for higher activity of *ARI* family members in the sexual MMC compared to the AIC.

In addition to miRNAs targeting F-box proteins and ARF transcription factors, miRNAs involved in regulation of SPL and MYB transcription factors have been identified in *Boechera* spp. [80]. Together with the enrichment observed for SPL transcription factors in the *B. gunnisoniana* AIC and of MYB transcription factors in the sexual *Arabidopsis* MMC and egg cell, respectively, this suggests that these transcription factors play important roles in plant reproduction. Differences in activity were also observed for additional transcription factor families in agreement with previously identified differences in transcriptional regulation at later developmental stages in sexual and apomictic *P. simplex* flowers [35]. In the sexual *Arabidopsis* egg cell as compared to the apomictic *Boechera* egg cell, we observed the enriched expression of the RKD transcription factor family, which are important regulators of egg cell gene expression programs in *Arabidopsis* and wheat [67]. This suggests that RKD transcription factors might be specifically involved in the determination of the developmental fate of the sexual egg cell. Taken together, our findings indicate differences in the activity of important regulatory pathways during sexual and apomictic germline specification and development.

### Germline specification during sexual reproduction and apomixis

Development of an unreduced embryo sac from an AIC is common to both diplospory and apospory. However, the founder cell of the female germline differs in position and cell fate between these two types of gametophytic apomixis. It is unknown whether a common regulator or a set of regulatory genes determines apomeiosis, or whether apomeiosis is mediated by unrelated developmental programs during apospory and diplospory. Interestingly, a number of important differences in gene expression have been observed in the aposporous initial cell in *H. praealtum* and the AIC of diplosporous *B. gunnisoniana*. This is consistent with the differences in cellular fate and identity between these apomicts. While the aposporous initial cell acquires a FMS-like fate without intervening cell division, the transcriptome of the AIC in a diplosporous apomict is expected to be more similar to the sexual MMC. This is in agreement with the lack of expression of several meiotic genes and other genes expressed in the sexual MMC in the aposporous initial cell in *H. praealtum* [31], differing from the transcriptome of the AIC in *Boechera*. Interestingly, in the *Boechera* AIC we did not observe evidence of expression of *DYAD*/*SWITCH*. In *Arabidopsis*, mutations in this gene have previously been shown to cause a diplospory-like phenotype with rare seed formation by the fertilization of unreduced egg cells [45]. The manipulation of cell cycle progression or meiotic genes has also been shown to lead to the formation of unreduced gametophytes [46], [83], [ reviewed in 84]. The comparison between the *Arabidopsis* MMC and the *Boechera* AIC identified a number of core cell cycle genes to be differentially regulated. While a small number of genes important for meiotic cell cycle progression in *Arabidopsis* has already been described [46], [ reviewed in 84], detailed functional studies of candidate genes showing differential expression in the MMC and AIC will be required to elucidate their putative role in the discrimination between meiosis and apomeiosis. Interestingly, the *Arabidopsis* gene encoding WEE1 is only detected in the *Arabidopsis* MMC. The WEE1 protein is specifically removed to allow progression of mitosis [85]. In addition, homologues of three members of the *Arabidopsis* E2F transcription factor family have only been detected in the *Boechera* AIC but not in the *Arabidopsis* MMC. Members of this family are involved in the regulation of the centromer-specific histone 3 variant CENH3 in *Arabidopsis* [86]. Manipulation of CENH3 can induce genome elimination, a capacity that has already been successfully applied for the generation of synthetic clonal seeds from *Arabidopsis* in combination with *dyad* or MiMe mutants [83]. Based on our transcriptome analysis, different levels of *CENH3* expression have been observed in the *Boechera* germline as compared to *Arabidopsis*. In contrast to very low expression or absence in *Arabidopsis* gametes, higher expression levels of the *CENH3* homologue have been observed in *Boechera* gametes. It is thus possible that the absence of *DYAD*/*SWITCH* expression in the AIC combined with elevated expression levels of CENH3 in apomictic *Boechera* as compared to sexual gametes might play a role in naturally occurring diplospory. In addition to unknown parthenogenesis factors, the regulation of CENH3 activity might provide an additional control mechanism to secure the absence of a paternal contribution in the offspring.

While mutations in the gene encoding for *DYAD* lead to features of diplospory, mutations in *MEM*, *AGO9* and additional genes involved in a small RNA pathway have recently been reported to cause phenotypes reminiscent of apospory [12], [42], [43]. We identified additional genes involved in gene silencing and small RNA pathways to be differentially expressed in the MMC and the AIC. The expression of *ESP3* in the AIC is reminiscent of the previous identification of *ESP4* among the transcripts form the apospory-specific region in *P. squamulatum* [87]. This supports the importance of epigenetic regulatory pathways for sexual and apomictic reproduction.

Taken together, upon specification of the apomictic and sexual germline a number of differences involving regulatory processes such as hormone signalling, cell cycle control, and protein turnover have been observed. In addition, increased activity of signal transduction processes was identified as a typical feature of the apomictic germline. The potential role of positioning of the MMC or AIC and the signalling from the surrounding sporophytic tissues has previously been discussed [88], [89], and our study has shown that signalling pathways are indeed modulated in the two modes of reproduction.

In conclusion, our study provides the first comprehensive transcriptional analysis of germline cells at key steps of apomictic reproduction in *B. gunnisoniana*. The generation and annotation of an apomictic reference transcriptome forms an essential basis for further analyses and allows the comparison of gene expression to *Arabidopsis* as sexual model species. Important differences in the development of the apomictic as compared to the sexual germline have been observed. While translational regulation is a feature conserved in both types of germline, polyamine and spermine/spermidine metabolism is only enriched upon initiation of the apomictic germline. In addition, key regulatory mechanisms are differentially regulated, involving hormone pathways, cell cycle control, signal transduction, and epigenetic regulatory processes. Thus, our analysis provides important new insights into gene regulation during apomictic germline development.

## Methods

### Plant material

*A. thaliana* Col-0 plants were used to isolate RNA for cloning of *in situ* probes. Plants were grown as described previously [12]. Seeds of *B. gunnisoniana* were kindly provided by Bitty Roy (University of Oregon, previously ETH Zürich) [48]. Seeds were surface sterilized and grown on MS plates for 10–14 days before transfer to a mixture of soil (ED73, Universalerde, Germany) and sand (5∶1), fertilized with Plantomaag (Syngenta, Basel, Switzerland) and Osmocote (Scotts, Marysville, USA). Plants were grown in a greenhouse chamber with 60% humidity and 16 h light/ 8 h darkness at 20°C and 16°C, respectively.

### Flow cytometry

Matured green seeds were harvested from *B. gunnisoniana* plants and individually analysed in a Quanta SC MPL flow cytometer (Beckman-Coulter, Nyon, Switzerland). Seeds were individually transferred to 1.2 ml cluster tubes (Thermo Scientific, Wohlen, Switzerland) containing 80 µl 0.1 M citric acid and 0.1% Triton X-100. A 3 mm stainless steel bead (Schieritz & Hauenstein AG, Zwingen, Switzerland) was added to each tube prior to shaking for 4 minutes at 30 Hz on a mixer mill (MM300, Retsch GmbH, Germany). Afterwards, 80 µl of 0.1 M citric acid containing 1% Triton X-100 was added and each tube was inverted 40 times. The solution containing the nuclei was filtered though fritted deep well plates (Nunc, Thermo Scientific, Wohlen, Switzerland) into 96-well V-bottom plates (Sarstedt, Numbrecht, Germany). Nuclei were collected by filtering in a centrifuge for 5 minutes at 150 g (Centrifuge 5810R, Eppendorf, Schönebuch, Switzerland). The nuclei were resuspended in 30 µl 0.1 M citric acid containing 1% Triton X-100. The samples were either analysed directly by flow cytometer robotics (Quanta SC MPL, Beckman-Coulter, Nyon, Switzerland) or stored at 4°C overnight prior to analysis. 120 µl of staining solution (0.4 M Na_2_HPO_4_, 2.6 ml H_2_O, 27.4 µl DAPI (5.5 µg/µl), 0.2 µl *β*-mercaptoethanol) were added 2 min prior to analysis. The protocol was set to count nuclei for six minutes or until a maximum of 10'000 counts was reached. The Photo Multiplier Tube and the gain were set to have the embryo peak at around 200 on the linear fluorescent scale. *B. stricta* nuclei were used as external standard.

### Cytological characterization

To quantitatively characterize developmental stages during germline development in *B. gunnisoniana*, material of 5 plants was used and averaged. Tissues were fixed in an ice-cold solution of ethanol∶acetic acid (3∶1; v/v), vacuum infiltrated on ice two times for 15 min, and left in fixative on ice over night before replacing the fixative with 70% ethanol. Tissues were cleared in chloral hydrate/glycerol/water (8∶1∶2; w/v/v), and microdissected with dissecting needles. Pictures were taken as previously described [12].

### *In situ* hybridization

Genes for data confirmation by *in situ* hybridization were selected based on the following criteria: (1) expression in the *B. gunnisoniana* AIC and no evidence of expression in the *A. thaliana* MMC, (2) representing different expression levels (Table S5), (3) high homology only to the respective homologue in *B. gunnisoniana* (82–96% identity between *A. thaliana* and *B. gunnisoniana* nucleotide sequences; Figure S3; Supporting Information S1), and (4) gene specificity in *A. thaliana*. Total RNA was isolated from *Arabidopsis* Col-0 inflorescences and from *B. gunnisoniana* buds and opened flowers using the RNeasy Plant Mini Kit (QIAGEN, Hilden, Germany). During the isolation procedure, RNA was treated with DNAseI on column. Reverse transcription was done as previously described ([12]; see Table S13 for a summary of primers and cDNA templates used). Fragment cloning and *in situ* hybridizations were done as previously described with modifications [12], [51], [90]: *in situ* hybridizations were performed on 8 µm thick sections of fixed and embedded *Boechera* buds or flowers. Pictures were taken and processed as previously described [12].

### Laser-assisted microdissection

To prepare samples for LAM, buds with ovules harbouring the AIC were chosen as previously described for selection of buds with ovules harbouring the MMC in *Arabidopsis* [12] with modifications: for *Boechera* individual buds were harvested instead of inflorescences. To obtain ovules harbouring mature gametophytes, flowers were emasculated ∼7 hours prior to fixation. The buds and flowers were fixed on ice in farmer's fixative (ethanol∶acetic acid 3∶1; v/v), vacuum infiltrated on ice two times for 15 min, and stored on ice over night before replacing the fixative with 70% ethanol. Embedding, microdissection, and LAM were done as previously described [12]. On average ∼60 sections of AICs were collected per day, or ∼25 sections for each cell-type of the mature female gametophyte. Egg and synergid cells from *Arabidopsis* were isolated as described previously for the central cell of *Arabidopsis* [13].

### RNA isolation and quality control

LAM samples were stored dry at −80°C before RNA isolation. RNA isolation and quality control was done as previously described [12], [13].

### Array hybridization

RNA amplification and labelling was done with the MessageAmpII Kit (Ambion, Foster City, USA) as described previously. ∼15 mg labeled aaRNA was fragmented and hybridized onto the *Arabidopsis* ATH1 GeneChip (Affymetrix) for 16 h at 45°C as described in the technical manual. The hybridization, staining, washing, and subsequent array scanning were performed as described previously [12]. Original data files are deposited under the Gene Expression Omnibus at NCBI (Accession Number GSE51996).

### SOLiD sequencing

RNA isolation, amplification, library preparation, and SOLiD Sequencing were performed as described previously [12], except that SOLiD V4 was used for paired-end sequencing. Original data files are deposited in the NCBI database (Accession Number: SRP032961).

### Reference transcriptome

As a tool for our data analysis we generated a reference transcriptome from female reproductive tissues of *B. gunnisoniana* at the two developmental stages of interest: (I) at megasporogenesis and (II) at the mature gametophyte stage. After isolation of mRNA and library preparation, sequencing was performed on an Illumina HiSeq 2000 instrument (see Supporting Methods S1 for details). Original data files are deposited in the NCBI SRA database (Accession Number SRP032960). The Transcriptome Shotgun Assembly project has been deposited at DDBJ/EMBL/GenBank under the accession GBAD00000000. The version described in this paper is the first version, GBAD01000000.

### Blast2GO annotation of the *B. gunnisoniana* reference transcriptome

After quality filtering, pre-processed reads were assembled using Trinity (version r2012-06-08) with default parameter settings, except that min_kmer_cov was set to 2. For annotation with Blast2GO, trinity assembled transcripts were compared to the NCBI non-redundant protein database (nr) using blastx (in blastall version 2.2.21). E-value cutoff was set to 0.00001. Top five hits were recorded. BLASTX results in XML format were analysed using b2g4pipe (version 2.5, [53]) to assign GO terms to the query transcript sequences.

### BLAT comparison of the *B. gunnisoniana* reference transcriptome to TAIR10 cDNA

The BLAT (version 34) comparison of the *Boechera* reference transcriptome and the TAIR10 cDNA sequences (updated 12/14/2010) was done with default parameters for cross species DNA mapping (-q = dnax -t = dnax). The top hits were selected using the blat utility script pslCDnafilter (globalNearBest, globalNearBest plus minCov of 80%). TAIR10 cDNA annotation of the top hits was then transferred to the query transcripts.

### Mapping of SOLiD reads

To obtain expression values based on the assembled *Boechera* reference transcriptome, short read data was processed as described in [13]. Gene-wise expression values were then defined as the sum of the expression values of individual transcript variants. Expression values based on the *A. thaliana* reference genome (TAIR10) were likewise calculated as described in [13].

### Defining closest *Arabidopsis* homologues for *Boechera* gene models

To identify potential homologues of known genes from *A. thaliana* in the assembled reference transcriptome of *B. gunnisoniana* we used BLAT (version 34, [54]). Sequences from *Boechera* were aligned to *Arabidopsis* cDNAs (TAIR10), allowing for a maximal intron size (-maxIntron) of 2 kb. Individual alignment scores (bitScore) and lengths between a given pair of *Boechera* and *Arabidopsis* sequences were then summed up. For each gene of interest from *Arabidopsis*, the *Boechera* homologue was then defined as the gene with the highest bitScore sum (or none if no alignments were reported or the total alignment length was below 100 bp).

### Analysis of sequence divergence

To estimate the extent of sequence divergence between a certain set of genes from *A. thaliana* and *B. gunnisoniana* we used ClustalX (version 2.1, [91]) with default settings (complete alignment, draw tree). Tree files were then used to cluster the genes in the heatmap plots (R packages ape, version 3.0-8 [92] and gplots, version 2.11.0, cran.r-project.org/web/packages/gplots/index.html).

### *Bg*PANP

Microarray data were processed as described in [12], except using an updated annotation of the ATH1 microarray (brainarray.mbni.med.umich.edu, TAIRG, version 14), and an alternative list of probesets for the background estimation (“negative probes”). Probe sequences were aligned to the assembled *Boechera* reference transcriptome using bowtie (version 0.12.7, [93]), allowing three mismatches. Probes without any alignments were considered as “negative probe” for the PANP algorithm [11].

### Gene set enrichment studies using NOISeq and EdgeR

We used the NOISeq-sim algorithm (downloaded in April 2012, http://bioinfo.cipf.es/noiseq/doku.php, [57]) to analyse differential expression of genes between RNA-Seq samples of the *Boechera* germline (apo_initial3, egg_cell2, central_cell2, synergid_cell2). Reads were aligned to the *Boechera* reference transcriptome. The normalization method was set to tmm (Trimmed mean of M, [94]), no correction for feature length was applied, and default settings were used for all other parameters, including q = 0.9 as threshold to determine differentially expressed genes. Genes identified as significantly upregulated in all three pairwise comparisons of one cell type with the other three *Boechera* germline samples were described as enriched in the cell type. For higher stringency analysis EdgeR was used with the biological coefficient of variation (bcv) set to 0.8 and Benjamini-Hochberg multiple testing corrections. Genes with an adjusted p value (FDR) below 0.05 were considered to be significantly differentially expressed. To identify genes differentially expressed between egg cell and central cell we applied an unadjusted p value<0.001.

### Identification of genes with evidence of expression only in *Boechera* or *Arabidopsis*

See Supporting Methods S1.

### Mappings

Gene Ontology (GO) terms associated with *A. thaliana* genes were extracted from the functional descriptions and GOSLIM mappings available on TAIR (ftp.arabidopsis.org/home/tair/Proteins/TAIR10_functional_descriptions, and (ftp.arabidopsis.org/home/tair/Ontologies/Gene_Ontology/ATH_GO_GOSLIM.txt). GO terms associated with the genes of *Boechera* were obtained with b2g4pipe (version 2.5, [54]). Protein family (PFAM) and gene family (FAM) annotation was used as described [95].

### GO, PFAM and FAM analyses

We used the Bioconductor package topGO [96] for gene ontology analysis. To test for overrepresentation of GO terms we used a Fisher's exact test in combination with the function “weight”. As gene universe in the test for *Arabidopsis* MMC the whole ATH1 array genome was used, otherwise all genes annotated in the respective GO annotation were used. We used a two-sided Fisher's exact test and comparison against the gene universe as defined above to test for misrepresentation of protein family domains (PFAM) and gene families (FAM).

### Heatmap clustering

Heatmaps were generated using the Bioconductor package gplots [97]. Hierachical agglomerative clustering (complete linkage) and euclidean distance was used. Normalization of RNA-Seq reads was done with the Bioconductor package DESeq [98]. Heatmaps were based on normalized log2-transformed total read counts for RNA-Seq data or log2-scale expression values generated by RMA for microarray data as previously described [12].

### Venn diagrams

Venn diagrams were made with the online tool BioVenn (http://www.cmbi.ru.nl/cdd/biovenn/).

## Supporting Information

Figure S1 Cytological characterization of reproductive development in *B. gunnisoniana*. (A, B) Development of the *B. gunnisoniana* AIC. A low percentage of AICs did not seem to divide (C) and likely arrested their development (D). (E) Dyad, (F) dyad with enlarged parietal cell or triad, (G) tetrad (artificially coloured in blue; based on the development of the integuments this tetrad is likely arrested or developmentally delayed), and (H) functional megaspore (FMS). (I, J) Mature gametophytes with unfused and fused polar nuclei, respectively. (K) Rarely more than one female gametophyte (artificially coloured in blue and pink) developed. (L–P) Seed development in young siliques after fertilization with embryo and endosperm development (L, M), young embryo developing in the absence of endosperm development (N), endosperm development without embryo development (O), and seed coat development in the absence of embryo or endosperm development (P). Black arrows point to AICs, stars mark (putative) parietal cells, white arrows point to dyads (or potential triads). Abbreviations: cen, central cell; egg, egg cell; syn, synergid cells; PN, polar nuclei; emb, embryo; end, endosperm. Scale bars are 40 µm. (Q) Summary of megaspore formation in *B. gunnisoniana*. In total 224 ovules were analysed. (R) Summary of mature gametophyte development in *B. gunnisoniana*. The percentages of mature gametophytes, gametophytes arrested at early developmental stages, gametophytes with an unexpected number of nuclei, and double gametophytes are given as analysed in 353 ovules.
(TIF)

Figure S2 Transcriptome analysis of the *Boechera* female gametes isolated by laser-assisted microdissection. (A) 6 µm thin section of a *Boechera* ovule harbouring the mature female gametophyte composed of egg cell, central cell, and synergid cells. Scale bar is 20 µm. (B, C) Venn diagrams showing the overlap of predicted expression in the *Boechera* and *Arabidopsis* female gametes. (B) Comparison of gene expression in the egg cell. Genes expressed in the *Arabidopsis* egg cell have been described before [11], [ reanalysed in 12] and were identified by RNA-Seq. Genes with evidence of expression in the *Boechera* egg cell were identified either by a P call with *Bg*PANP for the egg_cell1 sample or by at least 5 reads for homologues genes when mapped to the *Boechera* reference transcriptome. (C) Comparison of gene expression in the central cell. Genes expressed in the *Arabidopsis* central cell were previously identified using RNA-Seq [13]. Genes expression in the *Boechera* central cell was analysed by heterologous hybridization to the ATH1 microarray (central_cell1) or by RNA-Seq (central_cell2) by mapping the reads to the *Boechera* reference transcriptome and identification of *Arabidopsis* homologues.
(TIF)

Figure S3 Schematic alignment of *B. gunnisoniana* and *A. thaliana* genes selected for *in situ* hybridization. Schematic representation of gene exon and intron structures in *B. gunnisoniana* and *A. thaliana* for 5 genes selected for *in situ* hybridization. *Arabidopsis* gene and *Boechera* gene identifiers are given. The region selected for *in situ* probe design is indicated in red. Scaling is given in kb.
(TIF)

Figure S4 Heatmap of read counts for genes involved in silencing and small RNA pathways. Hierarchical clustering of log2 transformed read counts for 69 *Arabidopsis* genes homologues in *Boechera* involved in small RNA and gene silencing pathways (as used in [12]). RNA-Seq data from different *Arabidopsis* and *B. gunnisoniana* cell- and tissue types were used [13], [62]–[66]. The hierarchical clustering of samples and genes was based on euclidean distance and hierarchical agglomerative clustering. Colours are scaled per row. Red denotes high and black denotes low expression.
(TIF)

Figure S5 Heatmap of expression of *AGO* genes. (A) Hierarchical clustering of log2 transformed read counts of *AtAGO* genes and *Boechera* [13], [62]–[66]. (B) Hierarchical clustering of log2 scale expression values of *AtAGO* genes in *Arabidopsis* as analysed by the RMA algorithm [12]. (A, B) The hierarchical clustering of samples and genes was based on euclidean distance and hierarchical agglomerative clustering. Colours were scaled by row. Red denotes high and black low expression.
(TIF)

Figure S6 Analysis of sequence divergence and heatmap of expression. Analysis of sequence divergence of members of the *ARI* (A) and *AGO* (B) gene family and read counts assigned.
(TIF)

Figure S7 Heatmap of expression of *ARI* genes. (A) Hierarchical clustering of log2 transformed read counts of *AtARI* genes and *Boechera* homologues including datasets from different transcriptional studies [13], [62]–[66]. (B) Hierarchical clustering of log2 scale expression values of *AtARI* genes in *Arabidopsis* as analysed by the RMA algorithm [12]. The hierarchical clustering of samples and genes was based on euclidean distance and hierarchical agglomerative clustering. Colours were scaled by row. Red denotes high and black low expression.
(TIF)

Table S1 Annotation of *B. gunnisoniana* genes. *Boechera* genes were annotated using Blast2GO.
(ZIP)

Table S2 Assignment of *Arabidopsis* homologues to *Boechera* genes.
(TXT)

Table S3 *Bg*PANP expression calls. Datasheet with *Bg*PANP present (P) and absent (A) calls and p values in the AIC (apo_initial1, apo_initial2), the surrounding nucellus (sporo_nucellus1, and _2), egg cell (egg_cell1), central cell (central_cell), and synergid cells (synergid_cell) of *Boechera*.
(XLS)

Table S4 Expression values of individual variants of *Boechera* genes aligned to the reference transcriptome.
(TXT)

Table S5 Expression of genes selected for independent data confirmation by *in situ* analysis. P/A calls as analysed with *Bg*PANP for microarray samples and read counts for *B. gunnisoniana* homologues generated by mapping to the *B. gunnisoniana* reference transcriptome.
(PDF)

Table S6 Gene ontology (GO) analysis. (A) Molecular functions identified to be up-regulated based on 3'509 genes identified to be up-regulated in the egg cell as compared to the AIC, central cell and synergid cells. (B) Biological Processes identified to be up-regulated based on 1'806 genes identified to be up-regulated in the egg cell as compared to the AIC, central cell and synergid cells.
(PDF)

Table S7 Gene ontology (GO) analysis. Biological processes significantly upregulated in 142 genes enriched identified by EdgeR analysis in the *B. gunnisoniana* AIC as compared to the cell types of the mature female gametophyte.
(PDF)

Table S8 Analysis of protein family (PFAM) enrichment. Analysis of PFAM domains enriched in 852 genes with evidence of expression in the *Arabidopsis* MMC but not in the *B. gunnisoniana* AIC as tested by a two sided Fisher test. P values≤0.01 were considered significant.
(PDF)

Table S9 Analysis of protein family (PFAM) enrichment. Analysis of PFAM domains enriched in 2'146 genes with evidence of expression in the *Arabidopsis* but not in the *B. gunnisoniana* central cell as tested by a two sided Fisher test. P values≤0.01 were considered significant.
(PDF)

Table S10 Analysis of PFAM domains enriched in the apomictic *Boechera* germline. Significant enrichment of PFAM domains based on 901, 5'273 and 4'902 genes with evidence of expression in the AIC, egg cell and central cell of *Boechera* but not in the corresponding cell types of sexual *Arabidopsis* as analysed by two sided Fisher's exact test (p value<0.01).
(PDF)

Table S11 Enrichment of gene families in the *Boechera* female gametes. Significant enrichment of gene families based on 5'273 and 4'902 genes with evidence of expression in the egg cell and central cell of *Boechera* but not in the corresponding cell types of sexual *Arabidopsis* as analysed by two sided Fisher's exact test (p value<0.01).
(PDF)

Table S12 Analysis of expression of core cell cycle genes. Lists of core cell cycle genes only found to be expressed in the AIC or the MMC, but not other way round.
(XLS)

Table S13 Primers and templates used for cloning of *in situ* probes.
(PDF)

Methods S1 Methods description on the generation of the *B. gunnisoniana* reference transcriptome. Methods description on the identification of genes with evidence of expression only in *Boechera* or in *Arabidopsis*.
(PDF)

Supporting Information S1 Alignment of *in situ* probe sequences from *Arabidopsis* to the homologues *B. gunnisoniana* genes generated by BLAT [54].
(ZIP)

Supporting Information S2 Description of genes involved in the small RNA pathway or in the DNA methylation pathway only detected in *Arabidopsis* or in *Boechera* and description on the influence of sequence similarities between *Arabidopsis* and *Boechera* homologues on the distribution of count data. As examples the *ARI* and the *AGO* gene families are discussed.
(PDF)
