# Supplementary figures and images for: Apomictic and Sexual Germline Development Differ with Respect to Cell Cycle, Transcriptional, Hormonal and Epigenetic Regulation
Source: PLoS Genet. 2014 Jul 10;10(7):e1004476. doi: 10.1371/journal.pgen.1004476 (PMC4091798; doi:10.1371/journal.pgen.1004476)

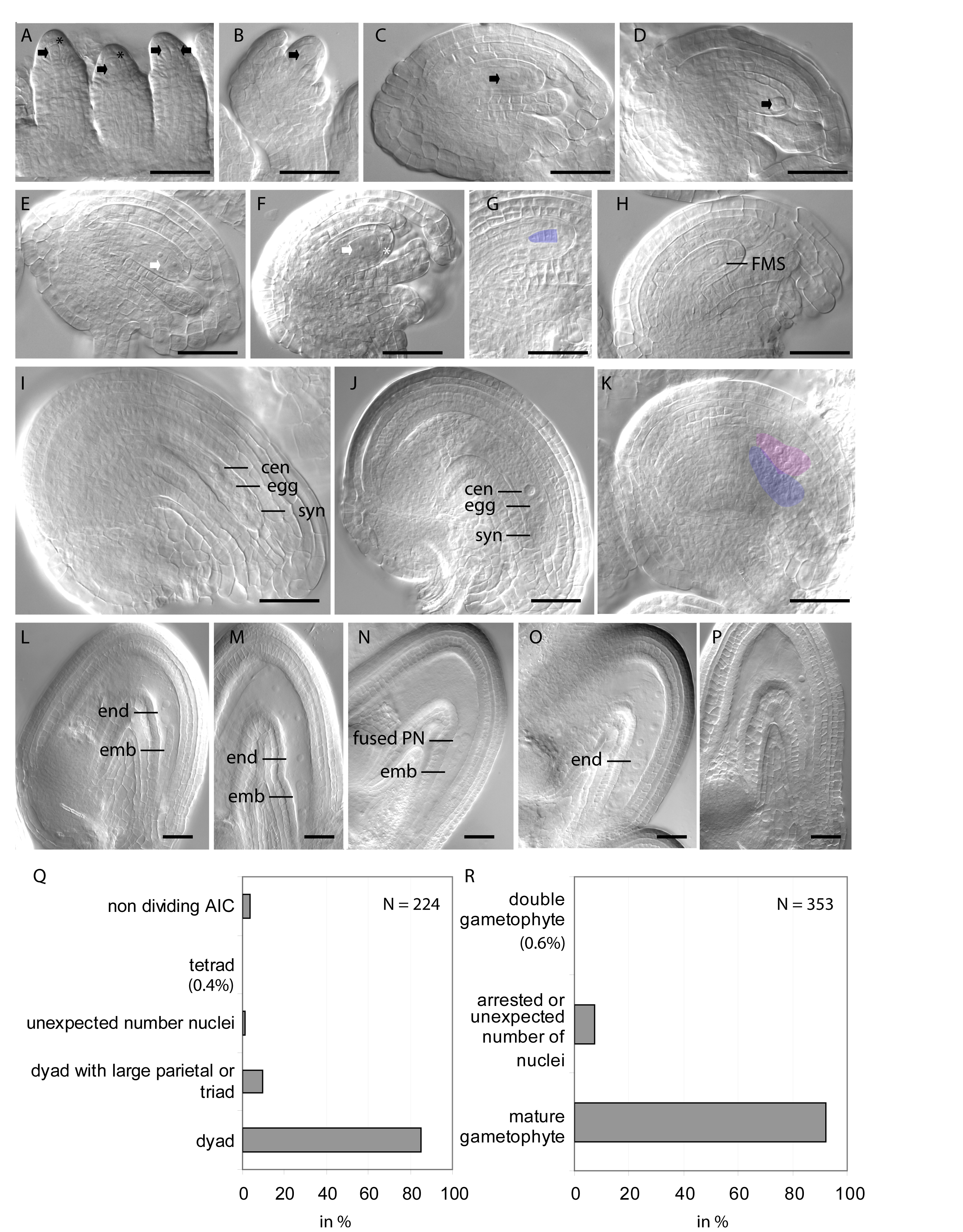

Supplement: Figure S1 — Cytological characterization of reproductive development in B. gunnisoniana. (A, B) Development of the B. gunnisoniana AIC. A low percentage of AICs did not seem to divide (C) and likely arrested their development (D). (E) Dyad, (F) dyad with enlarged parietal cell or triad, (G) tetrad (artificially coloured in blue; based on the development of the integuments this tetrad is likely arrested or developmentally delayed), and (H) functional megaspore (FMS). (I, J) Mature gametophytes with unfused and fused polar nuclei, respectively. (K) Rarely more than one female gametophyte (artificially coloured in blue and pink) developed. (L–P) Seed development in young siliques after fertilization with embryo and endosperm development (L, M), young embryo developing in the absence of endosperm development (N), endosperm development without embryo development (O), and seed coat development in the absence of embryo or endosperm development (P). Black arrows point to AICs, stars mark (putative) parietal cells, white arrows point to dyads (or potential triads). Abbreviations: cen, central cell; egg, egg cell; syn, synergid cells; PN, polar nuclei; emb, embryo; end, endosperm. Scale bars are 40 µm. (Q) Summary of megaspore formation in B. gunnisoniana. In total 224 ovules were analysed. (R) Summary of mature gametophyte development in B. gunnisoniana. The percentages of mature gametophytes, gametophytes arrested at early developmental stages, gametophytes with an unexpected number of nuclei, and double gametophytes are given as analysed in 353 ovules. (TIF) [file pgen.1004476.s001.tif]

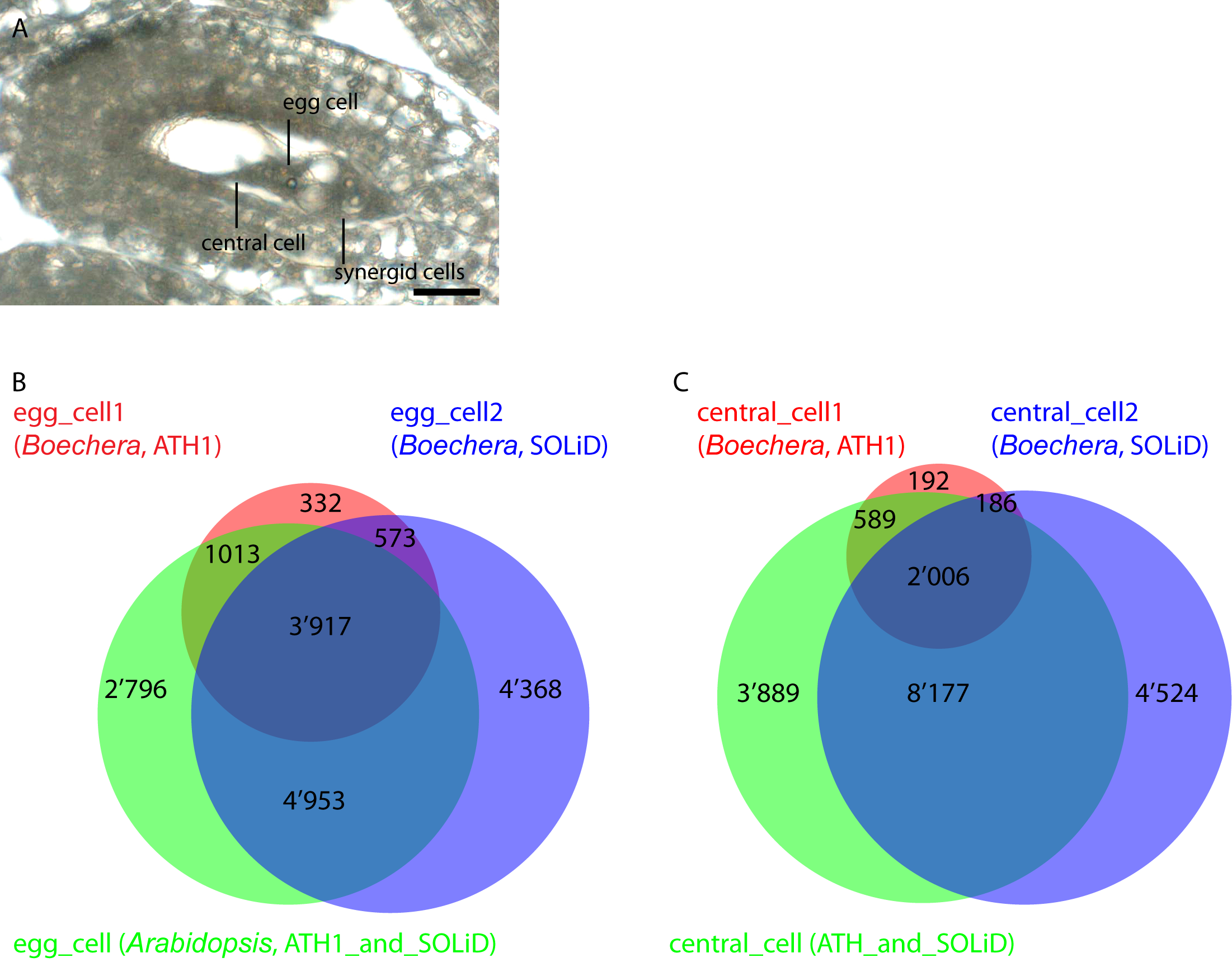

Supplement: Figure S2 — Transcriptome analysis of the Boechera female gametes isolated by laser-assisted microdissection. (A) 6 µm thin section of a Boechera ovule harbouring the mature female gametophyte composed of egg cell, central cell, and synergid cells. Scale bar is 20 µm. (B, C) Venn diagrams showing the overlap of predicted expression in the Boechera and Arabidopsis female gametes. (B) Comparison of gene expression in the egg cell. Genes expressed in the Arabidopsis egg cell have been described before [11], [ reanalysed in 12] and were identified by RNA-Seq. Genes with evidence of expression in the Boechera egg cell were identified either by a P call with BgPANP for the egg_cell1 sample or by at least 5 reads for homologues genes when mapped to the Boechera reference transcriptome. (C) Comparison of gene expression in the central cell. Genes expressed in the Arabidopsis central cell were previously identified using RNA-Seq [13]. Genes expression in the Boechera central cell was analysed by heterologous hybridization to the ATH1 microarray (central_cell1) or by RNA-Seq (central_cell2) by mapping the reads to the Boechera reference transcriptome and identification of Arabidopsis homologues. (TIF) [file pgen.1004476.s002.tif]

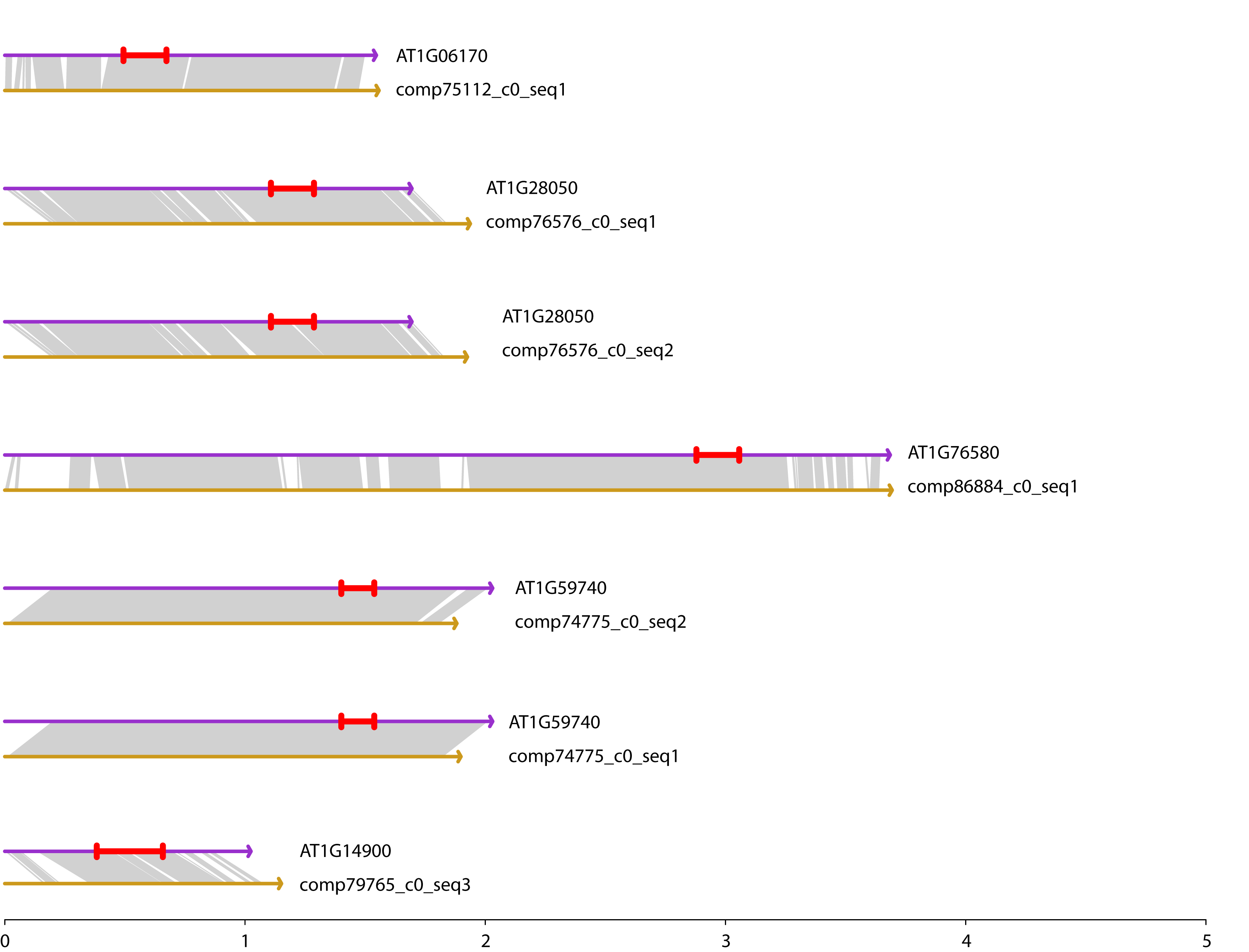

Supplement: Figure S3 — Schematic alignment of B. gunnisoniana and A. thaliana genes selected for in situ hybridization. Schematic representation of gene exon and intron structures in B. gunnisoniana and A. thaliana for 5 genes selected for in situ hybridization. Arabidopsis gene and Boechera gene identifiers are given. The region selected for in situ probe design is indicated in red. Scaling is given in kb. (TIF) [file pgen.1004476.s003.tif]

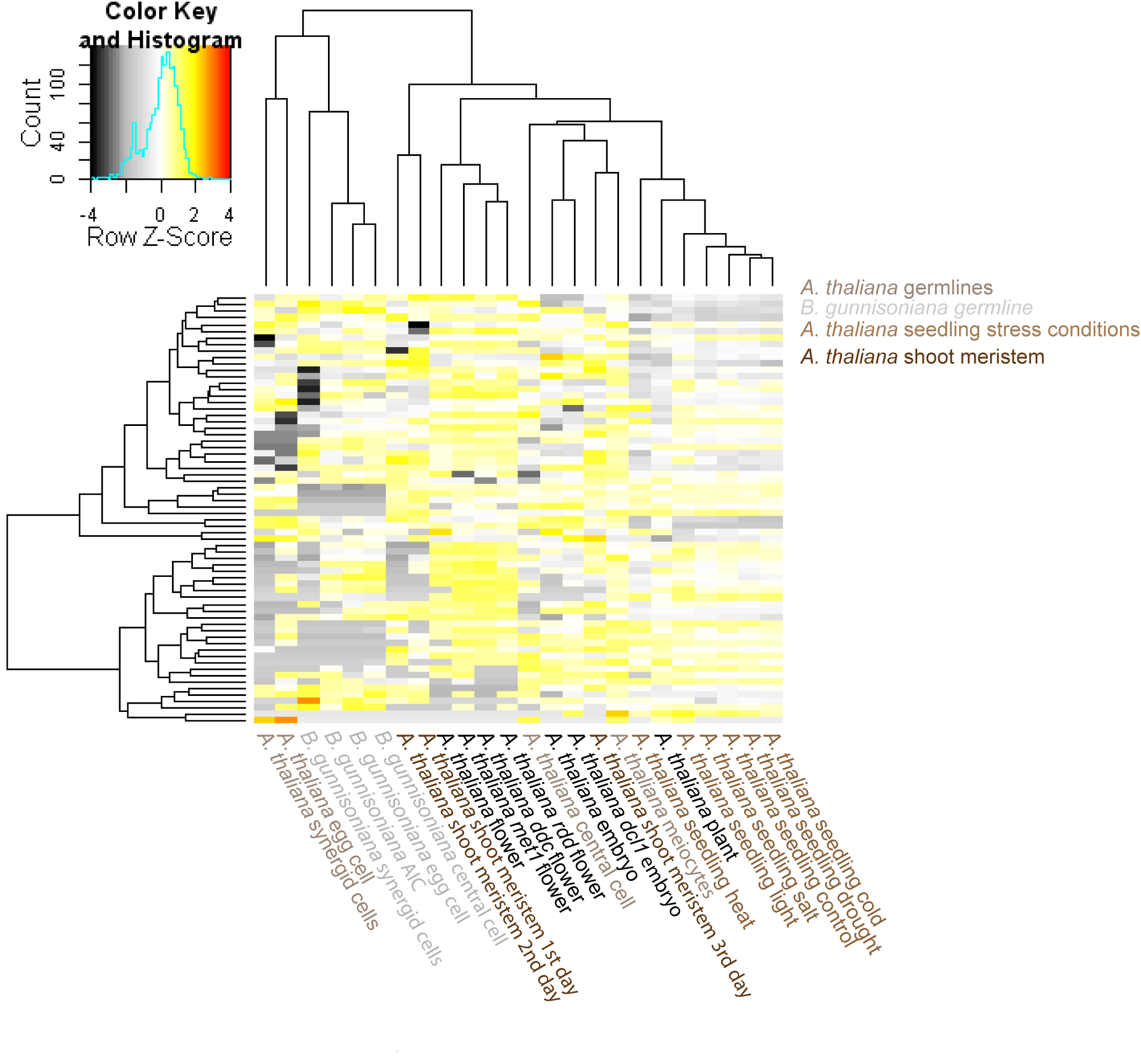

Supplement: Figure S4 — Heatmap of read counts for genes involved in silencing and small RNA pathways. Hierarchical clustering of log2 transformed read counts for 69 Arabidopsis genes homologues in Boechera involved in small RNA and gene silencing pathways (as used in [12]). RNA-Seq data from different Arabidopsis and B. gunnisoniana cell- and tissue types were used [13], [62]–[66]. The hierarchical clustering of samples and genes was based on euclidean distance and hierarchical agglomerative clustering. Colours are scaled per row. Red denotes high and black denotes low expression. (TIF) [file pgen.1004476.s004.tif]

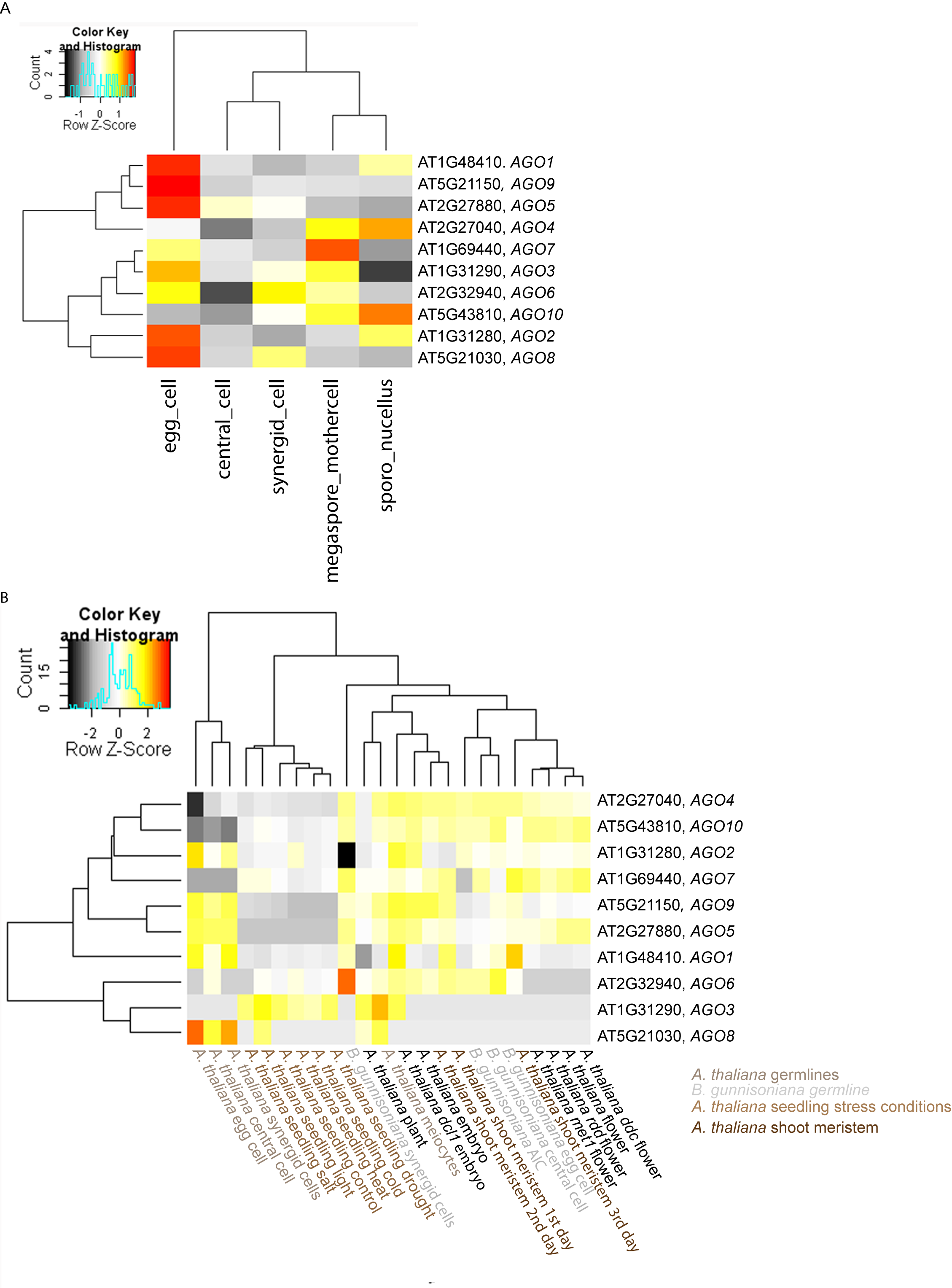

Supplement: Figure S5 — Heatmap of expression of AGO genes. (A) Hierarchical clustering of log2 transformed read counts of AtAGO genes and Boechera [13], [62]–[66]. (B) Hierarchical clustering of log2 scale expression values of AtAGO genes in Arabidopsis as analysed by the RMA algorithm [12]. (A, B) The hierarchical clustering of samples and genes was based on euclidean distance and hierarchical agglomerative clustering. Colours were scaled by row. Red denotes high and black low expression. (TIF) [file pgen.1004476.s005.tif]

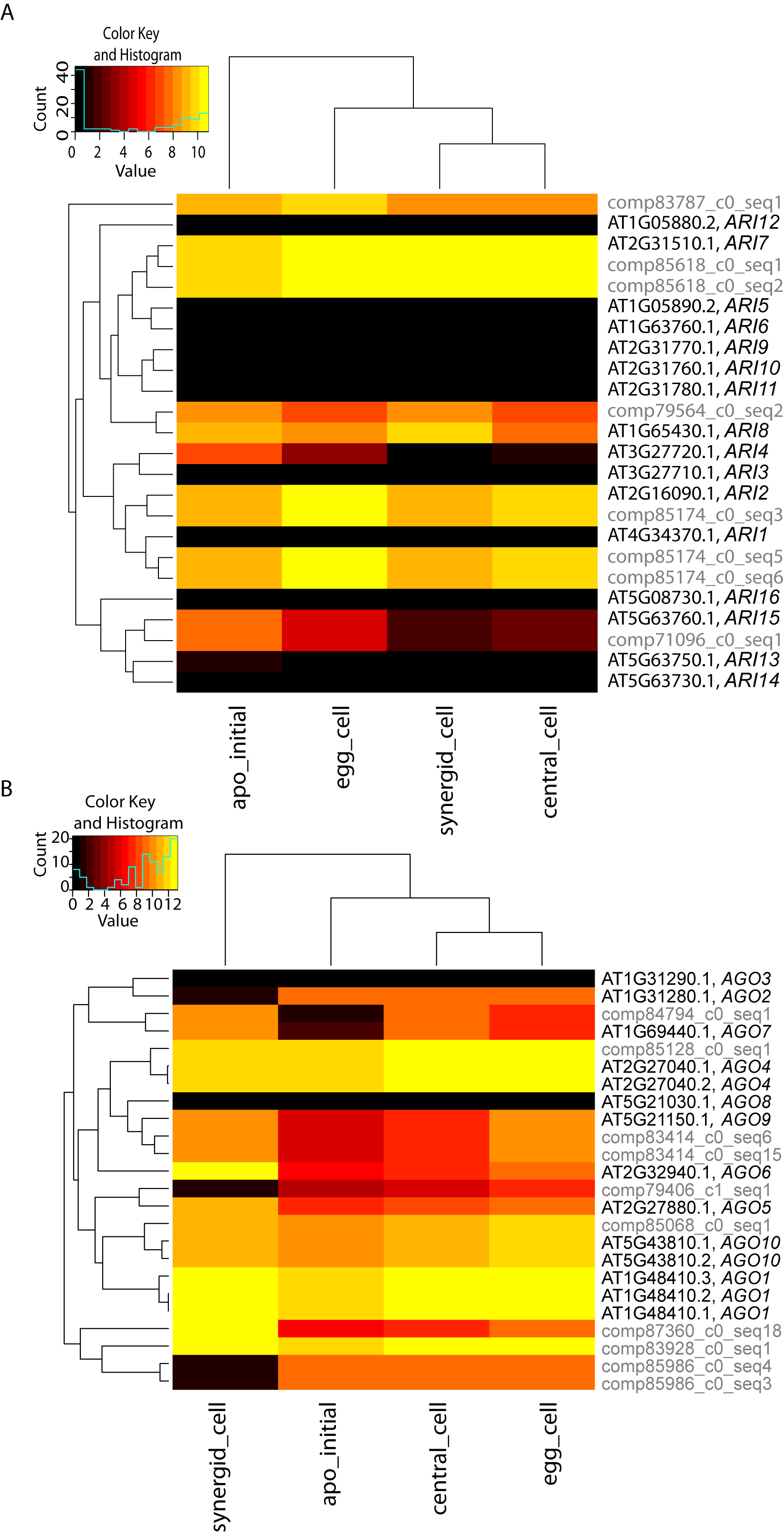

Supplement: Figure S6 — Analysis of sequence divergence and heatmap of expression. Analysis of sequence divergence of members of the ARI (A) and AGO (B) gene family and read counts assigned. (TIF) [file pgen.1004476.s006.tif]

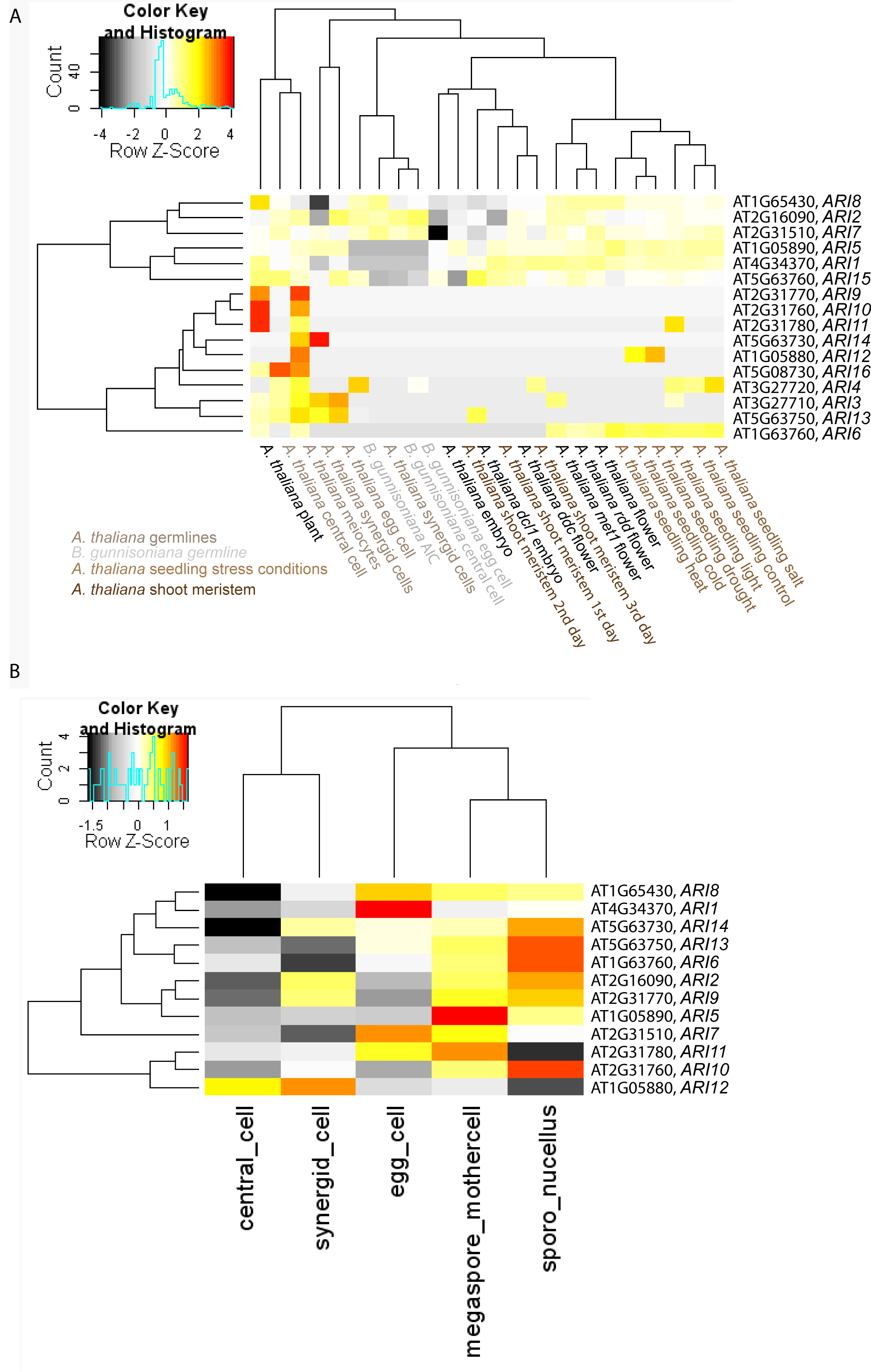

Supplement: Figure S7 — Heatmap of expression of ARI genes. (A) Hierarchical clustering of log2 transformed read counts of AtARI genes and Boechera homologues including datasets from different transcriptional studies [13], [62]–[66]. (B) Hierarchical clustering of log2 scale expression values of AtARI genes in Arabidopsis as analysed by the RMA algorithm [12]. The hierarchical clustering of samples and genes was based on euclidean distance and hierarchical agglomerative clustering. Colours were scaled by row. Red denotes high and black low expression. (TIF) [file pgen.1004476.s007.tif]
