# Supplementary material for: Apomictic and Sexual Germline Development Differ with Respect to Cell Cycle, Transcriptional, Hormonal and Epigenetic Regulation
Source: PLoS Genet. 2014 Jul 10;10(7):e1004476. doi: 10.1371/journal.pgen.1004476 (PMC4091798; doi:10.1371/journal.pgen.1004476)
Supplement: Table S5 — Expression of genes selected for independent data confirmation by in situ analysis. P/A calls as analysed with BgPANP for microarray samples and read counts for B. gunnisoniana homologues generated by mapping to the B. gunnisoniana reference transcriptome. (PDF) [file pgen.1004476.s012.pdf]

Table S5:

***Boechera* homologue to *Arabidopsis* gene**	**apo_initial1**	**apo_initial2**	**read counts in apo_initial3**	**sporo_nucellus1**	**sporo_nucellus2**
AT1G06170	A	A	4584	P	A
AT1G28050	P	P	322	P	P
AT1G76580	A	P	541	A	A
AT1G59740	P	P	21598	P	P
AT1G14900	A	A	1022	A	A
