# Supplementary material for: Apomictic and Sexual Germline Development Differ with Respect to Cell Cycle, Transcriptional, Hormonal and Epigenetic Regulation
Source: PLoS Genet. 2014 Jul 10;10(7):e1004476. doi: 10.1371/journal.pgen.1004476 (PMC4091798; doi:10.1371/journal.pgen.1004476)
Supplement: Table S6 — Gene ontology (GO) analysis. (A) Molecular functions identified to be up-regulated based on 3'509 genes identified to be up-regulated in the egg cell as compared to the AIC, central cell and synergid cells. (B) Biological Processes identified to be up-regulated based on 1'806 genes identified to be up-regulated in the egg cell as compared to the AIC, central cell and synergid cells. (PDF) [file pgen.1004476.s013.pdf]

Table S6:

A)

GO.ID	Term	Annotated	Significant	Expected	p value
GO:0016168	chlorophyll binding	27	18	3.42	9.70E-11
GO:0008266	poly(U) RNA binding	22	13	2.78	3.40E-07
GO:0003700	sequence-specific DNA binding transcription factor activity	1702	276	215.32	3.40E-06
GO:0015250	water channel activity	37	15	4.68	2.00E-05
GO:0005515	protein binding	3034	458	383.82	7.30E-05
GO:0003995	acyl-CoA dehydrogenase activity	8	6	1.01	9.10E-05
GO:0016620	oxidoreductase activity, acting on the aldehyde or oxo group of donors, NAD or NADP as acceptor	78	22	9.87	0.00019
GO:0080062	cytokinin 9-beta-glucosyltransferase activity	4	4	0.51	0.00026
GO:0010242	oxygen evolving activity	4	4	0.51	0.00026
GO:0047807	cytokinin 7-beta-glucosyltransferase activity	4	4	0.51	0.00026
GO:0051213	dioxygenase activity	153	35	19.36	0.00026
GO:0051537	2 iron, 2 sulfur cluster binding	36	13	4.55	0.00028
GO:0000976	transcription regulatory region sequence specific DNA binding	37	13	4.68	0.00038
GO:0050736	O-malonyltransferase activity	7	5	0.89	0.00054
GO:0016846	carbon-sulfur lyase activity	39	13	4.93	0.00068
GO:0050284	sinapate 1-glucosyltransferase activity	3	3	0.38	0.00202
GO:0003852	2-isopropylmalate synthase activity	3	3	0.38	0.00202
GO:0010294	abscisic acid glucosyltransferase activity	6	4	0.76	0.0031
GO:0009882	blue light photoreceptor activity	6	4	0.76	0.0031
GO:0003997	acyl-CoA oxidase activity	6	4	0.76	0.0031
GO:2001070	starch binding	10	5	1.27	0.00468
GO:0004930	G-protein coupled receptor activity	19	7	2.4	0.00647
GO:0004512	inositol-3-phosphate synthase activity	7	4	0.89	0.00651
GO:0004838	L-tyrosine:2-oxoglutarate aminotransferase activity	4	3	0.51	0.00732
GO:0005034	osmosensor activity	4	3	0.51	0.00732
GO:0001104	RNA polymerase II transcription cofactor activity	15	6	1.9	0.00736

B)

GO.ID	Term	Annotated	Significant	Expected	p value
GO:0006260	DNA replication	496	97	25.79	2.40E-30
GO:0000911	cytokinesis by cell plate formation	267	67	13.88	6.80E-28
GO:0034968	histone lysine methylation	318	73	16.54	9.60E-28
GO:0008283	cell proliferation	330	73	17.16	9.40E-26
GO:0051322	anaphase	162	47	8.42	7.60E-23
GO:0016458	gene silencing	506	73	26.31	3.80E-18
GO:0006334	nucleosome assembly	57	25	2.96	1.20E-17
GO:0006306	DNA methylation	237	48	12.32	3.20E-16
GO:0000086	G2/M transition of mitotic cell cycle	83	28	4.32	1.50E-14

GO:0048645	organ formation	255	46	13.26	6.00E-14
GO:0070925	organelle assembly	81	24	4.21	1.50E-12
GO:0001510	RNA methylation	240	39	12.48	2.10E-10
GO:0016572	histone phosphorylation	93	23	4.84	2.60E-10
GO:0000226	microtubule cytoskeleton organization	344	62	17.89	2.00E-09
GO:0009909	regulation of flower development	458	55	23.82	5.90E-09
GO:0006412	translation	636	66	33.07	8.50E-08
GO:0051726	regulation of cell cycle	321	60	16.69	1.20E-06
GO:0006302	double-strand break repair	133	22	6.92	4.30E-06
GO:0000280	nuclear division	132	22	6.86	8.90E-06
GO:0043570	maintenance of DNA repeat elements	5	4	0.26	3.50E-05
GO:0016444	somatic cell DNA recombination	39	9	2.03	0.00014
GO:0007018	microtubule-based movement	108	16	5.62	0.00014
GO:0000087	M phase of mitotic cell cycle	102	17	5.3	0.00019
GO:0000710	meiotic mismatch repair	8	4	0.42	0.00043
GO:0048509	regulation of meristem development	307	29	15.96	0.00111
GO:0009957	epidermal cell fate specification	25	6	1.3	0.00147
GO:0006338	chromatin remodeling	43	8	2.24	0.00148
GO:0009560	embryo sac egg cell differentiation	203	21	10.56	0.0021
GO:0016048	detection of temperature stimulus	2	2	0.1	0.0027
GO:0010440	stomatal lineage progression	62	9	3.22	0.00454
GO:0045910	negative regulation of DNA recombination	8	3	0.42	0.00644
GO:0001709	cell fate determination	8	3	0.42	0.00644
GO:0010212	response to ionizing radiation	117	13	6.08	0.00778
GO:0050982	detection of mechanical stimulus	3	2	0.16	0.00782
GO:0006777	Mo-molybdopterin cofactor biosynthetic process	16	4	0.83	0.008
GO:0006310	DNA recombination	339	41	17.63	0.00895
GO:0008356	asymmetric cell division	28	6	1.46	0.00989
