# Supplementary material for: Apomictic and Sexual Germline Development Differ with Respect to Cell Cycle, Transcriptional, Hormonal and Epigenetic Regulation
Source: PLoS Genet. 2014 Jul 10;10(7):e1004476. doi: 10.1371/journal.pgen.1004476 (PMC4091798; doi:10.1371/journal.pgen.1004476)
Supplement: Table S7 — Gene ontology (GO) analysis. Biological processes significantly upregulated in 142 genes enriched identified by EdgeR analysis in the B. gunnisoniana AIC as compared to the cell types of the mature female gametophyte. (PDF) [file pgen.1004476.s014.pdf]

Table S7:

GO.ID	Term	Annotated	Significant	Expected	p value
GO:0010584	pollen exine formation	92	28	0.56	< 1e-30
GO:0009830	cell wall modification involved in abscission	23	4	0.14	1.10E-05
GO:0009823	cytokinin catabolic process	10	3	0.06	2.60E-05
GO:0006629	lipid metabolic process	2163	28	13.18	2.70E-05
GO:0019953	sexual reproduction	337	10	2.05	7.40E-05
GO:0010876	lipid localization	276	8	1.68	0.00029
GO:0008216	spermidine metabolic process	25	3	0.15	0.00046
GO:0006817	phosphate ion transport	32	3	0.2	0.00096
GO:0080088	spermidine hydroxycinnamate conjugate biosynthetic process	1	1	0.01	0.00609
GO:0009859	pollen hydration	1	1	0.01	0.00609
