# Supplementary material for: Apomictic and Sexual Germline Development Differ with Respect to Cell Cycle, Transcriptional, Hormonal and Epigenetic Regulation
Source: PLoS Genet. 2014 Jul 10;10(7):e1004476. doi: 10.1371/journal.pgen.1004476 (PMC4091798; doi:10.1371/journal.pgen.1004476)
Supplement: Table S8 — Analysis of protein family (PFAM) enrichment. Analysis of PFAM domains enriched in 852 genes with evidence of expression in the Arabidopsis MMC but not in the B. gunnisoniana AIC as tested by a two sided Fisher test. P values≤0.01 were considered significant. (PDF) [file pgen.1004476.s015.pdf]

Table S8:

ID	Significant	Expected	p value	Family	Summary
PF00134	6	1.38891476	0.00447442	Cyclin_N	Cyclin, N-terminal domain
PF00646	45	10.0398696	1.13E-14	F-box	F-box domain
PF01344	11	3.05561248	0.00055495	Kelch_1	Kelch motif
PF01485	7	1.11113181	0.00031623	IBR	IBR domain
PF02984	6	1.07144853	0.0014315	Cyclin_C	Cyclin, C-terminal domain
PF03088	4	0.55556591	0.00426141	Str_synth	Strictosidine synthase
PF03145	4	0.67461574	0.00760569	Sina	Seven in absentia protein family
PF03478	5	1.15081509	0.00904619	DUF295	
PF04554	4	0.55556591	0.00426141	Extensin_2	Extensin like region
PF04776	5	0.51588263	0.00046358	DUF626	
PF05553	3	0.23809967	0.00395092	DUF761	Cotton fibre expressed protein
PF07723	16	2.18258034	9.83E-09	LRR_2	Leucine Rich Repeat
PF07734	11	2.93656265	0.00041107	FBA_1	F-box associated
PF08387	14	1.94448067	9.70E-08	FBD	
