# Supplementary material for: Apomictic and Sexual Germline Development Differ with Respect to Cell Cycle, Transcriptional, Hormonal and Epigenetic Regulation
Source: PLoS Genet. 2014 Jul 10;10(7):e1004476. doi: 10.1371/journal.pgen.1004476 (PMC4091798; doi:10.1371/journal.pgen.1004476)
Supplement: Table S9 — Analysis of protein family (PFAM) enrichment. Analysis of PFAM domains enriched in 2'146 genes with evidence of expression in the Arabidopsis but not in the B. gunnisoniana central cell as tested by a two sided Fisher test. P values≤0.01 were considered significant. (PDF) [file pgen.1004476.s016.pdf]

Table S9:

ID	Significant	Expected	p value	description
PF00646	76	39.5179534	1.15E-06	F-box domain
PF03080	14	3.73098212	0.00010954	DUF239
PF04043	21	9.36552654	0.0016098	PMEI
PF04554	6	1.21827988	0.00350033	Extensin_2
PF04776	6	1.52284984	0.00846085	DUF626
PF05617	30	3.12184218	1.92E-16	Prolamin-like
PF05938	30	5.02540448	2.31E-12	Self_incompS1
PF06915	6	1.44670735	0.00691689	DUF1278
PF07333	21	4.34012206	7.73E-08	SLR1_BP
PF07723	31	9.89852399	3.60E-07	LRR_2
PF07734	24	11.8020863	0.0029914	F-box associated
PF08387	30	8.7563866	1.07E-07	FBD
