# Supplementary material for: Apomictic and Sexual Germline Development Differ with Respect to Cell Cycle, Transcriptional, Hormonal and Epigenetic Regulation
Source: PLoS Genet. 2014 Jul 10;10(7):e1004476. doi: 10.1371/journal.pgen.1004476 (PMC4091798; doi:10.1371/journal.pgen.1004476)
Supplement: Table S10 — Analysis of PFAM domains enriched in the apomictic Boechera germline. Significant enrichment of PFAM domains based on 901, 5'273 and 4'902 genes with evidence of expression in the AIC, egg cell and central cell of Boechera but not in the corresponding cell types of sexual Arabidopsis as analysed by two sided Fisher's exact test (p value<0.01). (PDF) [file pgen.1004476.s017.pdf]

## Table S10:

**apomictic initial cell**

| ID | Significant | Expected | p value | description |
|---|---|---|---|---|
| PF00324 | 5 | 0.629483 | 0.00100098 | Amino acid permease |
| PF01490 | 9 | 1.6366558 | 0.00011589 | Transmembrane amino acid transporter protein |
| PF02309 | 7 | 1.84648347 | 0.00427111 | Aux/IAA family |
| PF03088 | 4 | 0.58751747 | 0.00515508 | Strictosidine synthase |
| PF03110 | 4 | 0.58751747 | 0.00515508 | SBP domain |
| PF04674 | 3 | 0.33572427 | 0.00849502 | Phosphate-induced protein 1 conserved region |
| PF06507 | 6 | 0.79734513 | 0.0003965 | Auxin response factor |

**egg cell**

| ID | Significant | Expected | p value | description |
|---|---|---|---|---|
| PF00010 | 42 | 24.88 | 0.00476216 | Helix-loop-helix DNA-binding domain |
| PF00069 | 183 | 131.90 | 0.00016034 | Protein kinase domain |
| PF00560 | 131 | 73.15 | 6.30E-08 | Leucine rich repeat |
| PF00730 | 9 | 2.25 | 0.00288164 | HhH-GPD superfamily base excision DNA repair protein |
| PF00786 | 10 | 2.99 | 0.004385 | P21-Rho binding domain |
| PF00931 | 68 | 31.43 | 5.85E-07 | NB-ARC domain |
| PF01535 | 197 | 82.32 | 3.67E-21 | Pentacortico repeat |
| PF01582 | 42 | 24.88 | 0.00476216 | Toll Interleukin receptor |
| PF07646 | 12 | 3.74 | 0.00238746 | Kelch motif |
| PF07714 | 100 | 63.80 | 0.00017157 | Protein tyrosine kinase |
| PF07725 | 34 | 15.34 | 0.00027842 | Leucine rich repeat, LRR_3 |
| PF08263 | 75 | 39.10 | 5.75E-06 | Leucine rich repeat N-terminal domain |

**central cell**

| ID | Significant | Expected | p value | description |
|---|---|---|---|---|
| PF00010 | 48 | 26.1526398 | 0.00058139 | Helix-loop-helix DNA-binding domain |
| PF00069 | 222 | 138.628655 | 7.03E-09 | protein kinase domain |
| PF00076 | 23 | 48.1759154 | 0.00025011 | RNA recognition motiv |
| PF00225 | 26 | 11.9948198 | 0.00198709 | Kinesin motor domain |
| PF00560 | 137 | 76.8848283 | 4.57E-08 | Leucine rich repeat |
| PF00612 | 24 | 11.0116378 | 0.00227686 | IQ calmodolin binding domain |
| PF00931 | 64 | 33.0349134 | 2.48E-05 | NB-ARC domain |
| PF01486 | 19 | 8.06209197 | 0.00435678 | K-box domain |
| PF01535 | 191 | 86.5200114 | 1.47E-17 | Pentacorticopeptide repeat |
| PF01582 | 44 | 26.1526398 | 0.00422265 | Toll-interleukin receptor |
| PF02183 | 12 | 4.12936418 | 0.00722254 | Homeobox associated leucine zipper |
| PF02362 | 33 | 18.2871842 | 0.00530936 | B3 domain |
| PF02458 | 24 | 12.3880925 | 0.00850195 | Transferase family |
| PF03000 | 16 | 6.48900085 | 0.00559973 | NPH3-family |
| PF03514 | 17 | 6.48900085 | 0.00323279 | GRAS domain family |
| PF04570 | 11 | 3.14618223 | 0.00233681 | DUF581 |
| PF04770 | 13 | 3.34281862 | 0.00047255 | ZF-HD protein dimerisation region |
| PF04784 | 10 | 3.14618223 | 0.0059545 | DUF547 |
| PF07714 | 115 | 67.0530088 | 2.29E-06 | Tyrosine kinase |
| PF07725 | 36 | 16.1241839 | 0.00015028 | Leucine rich repeat, LRR3 |
| PF08263 | 84 | 41.0970054 | 2.01E-07 | Leucine rich repeat N-terminal domain |
| PF11721 | 16 | 6.29236446 | 0.005044 | Di-glucose binding with endoplasmatic reticulum |
