# Supplementary material for: Apomictic and Sexual Germline Development Differ with Respect to Cell Cycle, Transcriptional, Hormonal and Epigenetic Regulation
Source: PLoS Genet. 2014 Jul 10;10(7):e1004476. doi: 10.1371/journal.pgen.1004476 (PMC4091798; doi:10.1371/journal.pgen.1004476)
Supplement: Table S11 — Enrichment of gene families in the Boechera female gametes. Significant enrichment of gene families based on 5'273 and 4'902 genes with evidence of expression in the egg cell and central cell of Boechera but not in the corresponding cell types of sexual Arabidopsis as analysed by two sided Fisher's exact test (p value<0.01). (PDF) [file pgen.1004476.s018.pdf]

Table S11:

egg cell

Gene family	Significant	Expected	p value
basic Helix-Loop-Helix (bHLH) Transcription Factor	47	29.7476228	0.00930689
Receptor kinase-like protein family	85	56.5017741	0.00155731

central cell

Gene family	Significant	Expected	p value
B3_TFs	31	17.1073659	0.00603793
basic Helix-Loop-Helix (bHLH) Transcription Factor	65	31.2651859	3.04E-06
bHLH Transcription Factor Family	64	30.8719131	4.08E-06
GRAS Gene Family	16	6.29236446	0.005044
GRAS Transcription Factor Family	16	6.09572807	0.00271396
IQD Protein Family	20	6.09572807	8.66E-05
Kinesins	26	11.9948198	0.00198709
Receptor kinase-like protein family	110	59.3841896	1.60E-07
ZF-HD Transcription Factor Family	12	2.94954584	0.00058824
zinc finger-homeobox gene family	13	3.34281862	0.00047255
