# Supplementary material for: Apomictic and Sexual Germline Development Differ with Respect to Cell Cycle, Transcriptional, Hormonal and Epigenetic Regulation
Source: PLoS Genet. 2014 Jul 10;10(7):e1004476. doi: 10.1371/journal.pgen.1004476 (PMC4091798; doi:10.1371/journal.pgen.1004476)
Supplement: Table S13 — Primers and templates used for cloning of in situ probes. (PDF) [file pgen.1004476.s020.pdf]

Table S13: Primers and templates used for cloning of *in situ* probes.

gene	primer	template	sequence
AT1G06170	P1	Col-0 inflorescence cDNA	CCACTCCTGACCTACTCAGTC
AT1G06170	P2	Col-0 inflorescence cDNA	CTTGATCCACTCCCATGAAACTC
AT1G28050	P3	Col-0 inflorescence cDNA	GATGACTACAAGCGATCAACTTC
AT1G28050	P4	Col-0 inflorescence cDNA	CAGATCAGCCTTAGTTGCAACTAG
AT1G76580	P5	Col-0 inflorescence cDNA	ATGAGAGACAACCACAGTTACAAC
AT1G76580	P6	Col-0 inflorescence cDNA	CTCTGATACTTTAGAGCCACACTTG
AT1G59740	P7	Col-0 inflorescence cDNA	TGCACGTAAGCTCACAGGAC
AT1G59740	P8	Col-0 inflorescence cDNA	CCGTCAAGAACCGAAGAGTCTC
AT1G14900	P9	Col-0 inflorescence cDNA	CGTCCTCCGAAGCAGAAGAC
AT1G14900	P10	Col-0 inflorescence cDNA	CTTCGGAGGTCTCCCACGT
