# Supplementary material for: Apomictic and Sexual Germline Development Differ with Respect to Cell Cycle, Transcriptional, Hormonal and Epigenetic Regulation
Source: PLoS Genet. 2014 Jul 10;10(7):e1004476. doi: 10.1371/journal.pgen.1004476 (PMC4091798; doi:10.1371/journal.pgen.1004476)
Supplement: Methods S1 — Methods description on the generation of the B. gunnisoniana reference transcriptome. Methods description on the identification of genes with evidence of expression only in Boechera or in Arabidopsis. (PDF) [file pgen.1004476.s021.pdf]

Supporting Methods S1

Generation of a *B. gunnisoniana* Reference Transcriptome

Sample preparation: Buds and ovules of *B. gunnisoniana* were selected based on size and morphology. The samples contained slightly earlier or later developmental stages and additional pistil tissues in low amounts. We prepared one sample each for both developmental stages of interest. The ovules were microdissected using dissecting needles and snap frozen in liquid nitrogen. For each sample, ovule material from at least two individual plants and one to several buds per plant were pooled. Total RNA was isolated PicoPure RNA isolation kit (Arcturus Engineering, Mountenview, USA) following the manufacturer instructions with modifications. RNA was on column treated with DNAseI (QIAGEN, Hilden, Germany).

Library preparation: The quality of the isolated RNA was determined with a Qubit (1.0) Fluorometer (Life Technologies, California, USA) and a Bioanalyzer 2100 (Agilent, Waldbronn, Germany). Samples with a 260 nm/280 nm ratio between 1.8–2.1 and a 28S/18S ratio within 1.5–2 were further processed. The TruSeq RNA Sample Prep Kit v2 (Illumina, Inc, California, USA) was used in the succeeding steps. Briefly, total RNA samples (400-1000 ng) were poly A enriched and then reverse-transcribed into double-stranded cDNA. The cDNA samples was fragmented, end-repaired and polyadenylated before ligation of TruSeq adapters containing the index for multiplexing Fragments containing TruSeq adapters on both ends were selectively enriched with PCR. The quality and quantity of the enriched libraries were validated using Qubit (1.0) Fluorometer and the Caliper GX LabChip GX (Caliper Life Sciences, Inc., USA). The product is a smear with an average fragment size of approximately 260 bp. The libraries were normalized to 10nM in Tris-Cl 10 mM, pH 8.5 with 0.1% Tween 20.

Cluster Generation and Sequencing: The TruSeq PE Cluster Kit v3-cBot-HS (Illumina, Inc, California, USA) was used for cluster generation using 10 pM of pooled

normalized libraries on the cBOT. Sequencing was performed on the Illumina HiSeq 2000 paired end at 2 x 101 bp using the TruSeq SBS Kit v3-HS (Illumina, Inc, California, USA).

Filtering procedures: For quality filtering HiSeq raw reads were trimmed and filtered using prinseq-lite.pl (version 0.17.4). Bases on both ends with quality scores lower than Q20 were trimmed. Trimmed reads with average quality scores below Q30 or shorter than 25 bases were discarded. Sequencing adaptor contamination and other sequencing artefacts were screened using Tagdust (version 1.13) with default parameter setting.

Identification of Genes with Evidence of Expression only in *Boechera* or *Arabidopsis*

For selection of genes with evidence of expression in the *Arabidopsis* MMC but not in the *Boechera* AIC we selected all 9’115 genes with a P call on at least 2 out of 4 biological replicates as on the microarrays as expressed [1]. From this list we subtracted all genes with (i) a P call on both arrays hybridized with the apo_initial1 and apo_initial2 samples, (ii) at least 5 read counts for *Arabidopsis* homologues when mapped to the *Boechera* reference transcriptome, or (iii) at least 5 read counts when mapped to the *Arabidopsis* reference genome. We included the mapping to the *Arabidopsis* reference genome for a conservative estimate. However, mapping to the *Arabidopsis* reference transcriptome can lead to an underestimate of expression for certain genes, particularly if a subset of the sequences does not fulfil the mapping criteria due to sequence divergence between the *Arabidopsis* gene and the *Boechera* homologue. For identification of genes only expressed in the *Arabidopsis* female gametes, we summarized genes with a P call in at least 2 out of 3 biological replicates on the ATH1 microarray [2, reanalysed in 1] and at least 5 reads in the one or two RNA-Seq samples mapped to the *Arabidopsis* reference genome for egg- and central-cell, respectively. For a conservative estimate of genes expressed in the *Boechera* AIC but not in the *Arabidopsis* MMC we summarized all genes with more than 5 reads from the apo_initial3

sample supported by a P call with *Bg*PANP in apo_initial1 and/or apo_initial2 and subtracted all genes with evidence of expression in the *Arabidopsis* MMC as defined above. To identify genes with evidence of expression only in the apomictic *Boechera* egg cell or central cell we summarized the genes with a P call from our *Bg*PANP analysis of the mircoarray data (egg_cell1, central_cell2) with the genes with more than 5 reads in the RNA-Seq sample (via_comp) in egg_cell2 and central_cell2, respectively. From these datasets we subtracted all genes with evidence of expression in the respective cell type in *Arabidopsis*, as defined above.

References

1. Schmidt A, Wüst SE, Vijverberg K, Baroux C, Kleen D, et al. (2011) Transcriptome analysis of the *Arabidopsis* megaspore mother cell uncovers the importance of RNA helicases for plant germline development. PLoS Biol 9: e1001155.
2. Wüst SE, Vijverberg K, Schmidt A, Weiss M, Gheyselinck J, et al. (2010) *Arabidopsis* female gametophyte gene expression map reveals similiarities between plant and animal gametes. Curr Biol 20: 506-512.
