# Supplementary material for: Apomictic and Sexual Germline Development Differ with Respect to Cell Cycle, Transcriptional, Hormonal and Epigenetic Regulation
Source: PLoS Genet. 2014 Jul 10;10(7):e1004476. doi: 10.1371/journal.pgen.1004476 (PMC4091798; doi:10.1371/journal.pgen.1004476)
Supplement: Supporting Information S2 — Description of genes involved in the small RNA pathway or in the DNA methylation pathway only detected in Arabidopsis or in Boechera and description on the influence of sequence similarities between Arabidopsis and Boechera homologues on the distribution of count data. As examples the ARI and the AGO gene families are discussed. (PDF) [file pgen.1004476.s023.pdf]

Supporting Information S2

Genes involved in the small RNA pathway or in the DNA methylation pathway only detected in the sexual *Arabidopsis* MMC or the *Boechera gunnisoniana* AIC

From 69 genes involved in small RNA or DNA methylation pathways, evidence of expression in the MMC was given for a number of genes not detected in the AIC. For *DORMANCY-ASSOCIATED PROTEIN* 1 (*DYL1*), *RNA-DIRECTED DNA METHYLATION 1 (RDM1)*, *RIBOSOMAL RNA PROCESSING 4*, *REPRESSOR OF SILENCING 3* (*ROS3*), *SU*(*VAR*)*3-9 HOMOLOG 6* (*SUVH6*), and *DEFECTIVE IN MERISTEM SILENCING 3* no *Boechera* homologue has been annotated in our reference transcriptome. In contrast, a *DAWDLE* (*DDL*) homologue has been annotated in the *B. gunnisoniana* reference transcriptome and gene expression has only been observed in the MMC. *DDL* is involved in the biogenesis of small RNA [1]. Under stringent criteria also taking the mapping of *Boechera* reads against the *Arabidopsis* reference genome into account, only *DYL1*, *RDM1* ant *SUVH6* are likely to be expressed in the MMC but not in the AIC. In contrast, *RNA-DEPENDENT RNA POLYMERASE2* (*RDR2*), *FASCIATA1*, *ROS1*, *SUVH2*, *FLOWERING LOCUS D*, *DSRNA-BINDING PROTEIN5*, *HUA ENHANCER1* (*HEN1*) and *HEN2*, *DEMETER*, *DECREASED DNA METHYLATION1*, and *ENHANCED SILENCING PHENOTYPE3* (*ESP3*) have only been detected in the AIC. In addition, *NUCLEAR RNA POLYMERASE D 1A* has been detected in the AIC at very low levels (<50 reads), likely below the detection limit of the ATH1 microarray used for transcriptome profiling of the MMC.

Influence of sequence similarities between *Arabidopsis* and *Boechera* homologues on the distribution of count data

The *Arabidopsis ARI* gene family has 16 annotated members. For *Boechera* we identified 8 gene variants of 5 genes (Figure S6A). For example for comp71096_c0 *AtARI15* has been identified as closest homologue. However, for other *Boechera* genes different variants match

closest to different *Arabidopsis* genes. While comp85174_c0_seq5, and _seq6 cluster closest to *ARI1*, and comp85174_c0_seq3 to *ARI2*, reads from the *Boechera* gene model comp85174_c0 get assigned to *ARI2* only (Figure S6A). For *AtARI7*, the *Boechera* gene model comp85618_c0 is the closest homologue. Notably, the heatmap clustering of log2 transformed read counts of RNA-Seq data (Figure S6A) and the RMA expression values from microarray data from the *Arabidopsis* germline lineage (Figure S7) indicates the overall predominant expression of the *ARI* gene family during *Arabidopsis* sporogenesis.

The *AGO* gene family has 10 annotated members in *Arabidopsis*. From the *Boechera* reference transcriptome, 7 genes with homology to *AtAGO* genes have been identified (Figure S6B). *AGO4* clusters closest to the *Boechera* gene comp85128_c0, *AGO7* to comp84794_c0, *AGO9* to comp83414_c0, and *AGO10* to comp85068_c0, and reads are distributed accordingly. Other *Boechera* show more sequence divergence from their *Arabidopsis* homologues, for example comp87360_c0, comp83928_c0, and comp85986_c0 form a separate cluster.

Reference

1. Yu B, Bi L, Zheng B, Ji L, Chevalier D, et al. (2008) The FHA domain proteins DAWDLE in Arabidopsis and SNIP1 in humans act in small RNA biogenesis. Proc Natl Acad Sci U S A 105: 10073-8.
